# Complete prevalence and indicators of cancer cure: enhanced methods and validation in Italian population-based cancer registries

**DOI:** 10.3389/fonc.2023.1168325

**Published:** 2023-06-06

**Authors:** Federica Toffolutti, Stefano Guzzinati, Angela De Paoli, Silvia Francisci, Roberta De Angelis, Emanuele Crocetti, Laura Botta, Silvia Rossi, Sandra Mallone, Manuel Zorzi, Gianfranco Manneschi, Ettore Bidoli, Alessandra Ravaioli, Francesco Cuccaro, Enrica Migliore, Antonella Puppo, Margherita Ferrante, Cinzia Gasparotti, Maria Gambino, Giuliano Carrozzi, Fabrizio Stracci, Maria Michiara, Rossella Cavallo, Walter Mazzucco, Mario Fusco, Paola Ballotari, Giuseppe Sampietro, Stefano Ferretti, Lucia Mangone, Roberto Vito Rizzello, Michael Mian, Giuseppe Cascone, Lorenza Boschetti, Rocco Galasso, Daniela Piras, Maria Teresa Pesce, Francesca Bella, Pietro Seghini, Anna Clara Fanetti, Pasquala Pinna, Diego Serraino, Luigino Dal Maso, AIRTUM Working Group, Fabiola Giudici

**Affiliations:** ^1^ Cancer Epidemiology Unit, Centro di Riferimento Oncologico di Aviano (CRO) Istituto di Ricovero e Cura a Carattere Scientifico (IRCCS), Aviano, Italy; ^2^ Epidemiological Department, Azienda Zero, Padua, Italy; ^3^ National Centre for Disease Prevention and Health Promotion, National Institute of Health, Rome, Italy; ^4^ Department of Oncology and Molecular Medicine, National Institute of Health, Rome, Italy; ^5^ Evaluative Epidemiology Unit, Department of Research, Fondazione IRCCS Istituto Nazionale dei Tumori di Milano, Milan, Italy; ^6^ Tuscany Cancer Registry, Clinical Epidemiology Unit, Institute for Cancer Research, Prevention and Clinical Network (ISPRO), Florence, Italy; ^7^ Emilia-Romagna Cancer Registry, Romagna Unit, IRCCS Istituto Romagnolo per lo Studio dei Tumori (IRST) “Dino Amadori”, Forlì, Italy; ^8^ Registro Tumori Puglia - Sezione Azienda Sanitaria Locale (ASL) Barletta-Andria-Trani, Epidemiologia e Statistica, Barletta, Italy; ^9^ Piedmont Cancer Registry, Centro di Riferimento per l'Epidemiologia e la Prevenzione Oncologica (CPO) Piemonte and University of Turin, Turin, Italy; ^10^ Liguria Cancer Registry, IRCCS Ospedale Policlinico San Martino, Genova, Italy; ^11^ Registro tumori integrato di Catania-Messina-Enna, Igiene Ospedaliera, Azienda Ospedaliero-Universitaria Policlinico G. Rodolico-San Marco, Catania, Italy; ^12^ Struttura Semplice Epidemiologia, Agenzia di Tutela della Salute (ATS) Brescia, Brescia, Italy; ^13^ Registro tumori ATS Insubria (Provincia di Como e Varese) Responsabile S.S. Epidemiologia Registri Specializzati e Reti di Patologia, Varese, Italy; ^14^ Emilia-Romagna Cancer Registry, Modena Unit, Public Health Department, Local Health Authority, Modena, Italy; ^15^ Umbria Cancer Registry, Public Health Section, Department of Medicine and Surgery, University of Perugia, Perugia, Italy; ^16^ Emilia-Romagna Cancer Registry, Parma Unit, Medical Oncology Unit, University Hospital of Parma, Parma, Italy; ^17^ Cancer Registry Azienda Sanitaria Locale (ASL) Salerno- Dipartimento di Prevenzione, Salerno, Italy; ^18^ Clinical Epidemiology and Cancer Registry Unit, Azienda Ospedaliera Universitaria Policlinico (AOUP) di Palermo, Palermo, Italy; ^19^ Registro Tumori ASL Napoli 3 Sud, Napoli, Italy; ^20^ Osservatorio Epidemiologico, ATS Val Padana, Mantova, Italy; ^21^ Servizio Epidemiologico ATS di Bergamo, Bergamo, Italy; ^22^ Emilia-Romagna Cancer Registry, Ferrara Unit, Local Health Authority, Ferrara, and University of Ferrara, Ferrara, Italy; ^23^ Emilia-Romagna Cancer Registry, Reggio Emilia Unit, Epidemiology Unit, Azienda Unità Sanitaria Locale - IRCCS di Reggio Emilia, Reggio Emilia, Italy; ^24^ Trento Province Cancer Registry, Unit of Clinical Epidemiology, Trento, Italy; ^25^ Innovation, Research and Teaching Service (SABES-ASDAA), Lehrkrankenhaus der Paracelsus Medizinischen Privatuniversität, Bolzano-Bozen, Italy; ^26^ Azienda Sanitaria Provinciale (ASP) Ragusa - Dipartimento di Prevenzione -Registro Tumori, Ragusa, Italy; ^27^ Cancer Registry of the Province of Pavia, Pavia, Italy; ^28^ Unit of Regional Cancer Registry, Clinical Epidemiology and Biostatistics, IRCCS Centro di Riferimento Oncologico di Basilicata (CROB), Rionero in Vulture, Italy; ^29^ Nord Sardegna Cancer Registry, ASL, Sassari, Italy; ^30^ Monitoraggio rischio ambientale e Registro Tumori ASL Caserta, Caserta, Italy; ^31^ Siracusa Cancer Registry, Provincial Health Authority of Siracusa, Siracusa, Italy; ^32^ Emilia-Romagna Cancer Registry, Piacenza Unit, Public Health Department, AUSL Piacenza, Piacenza, Italy; ^33^ Sondrio Cancer Registry, Agenzia di Tutela della Salute della Montagna, Sondrio, Italy; ^34^ Nuoro Cancer Registry, RT Nuoro, Servizio Igiene e Sanità Pubblica, ASL Nuoro, Nuoro, Italy

**Keywords:** prevalence, cancer cure indicators, time to cure, Italy, survival, cure fraction, cure prevalence

## Abstract

**Objectives:**

To describe the procedures to derive complete prevalence and several indicators of cancer cure from population-based cancer registries.

**Materials and methods:**

Cancer registry data (47% of the Italian population) were used to calculate limited duration prevalence for 62 cancer types by sex and registry. The incidence and survival models, needed to calculate the completeness index (*R*) and complete prevalence, were evaluated by likelihood ratio tests and by visual comparison. A sensitivity analysis was conducted to explore the effect on the complete prevalence of using different *R* indexes. Mixture cure models were used to estimate net survival (NS); life expectancy of fatal (LEF) cases; cure fraction (CF); time to cure (TTC); cure prevalence, prevalent patients who were not at risk of dying as a result of cancer; and already cured patients, those living longer than TTC at a specific point in time. CF was also compared with long-term NS since, for patients diagnosed after a certain age, CF (representing asymptotical values of NS) is reached far beyond the patient’s life expectancy.

**Results:**

For the most frequent cancer types, the Weibull survival model stratified by sex and age showed a very good fit with observed survival. For men diagnosed with any cancer type at age 65–74 years, CF was 41%, while the NS was 49% until age 100 and 50% until age 90. In women, similar differences emerged for patients with any cancer type or with breast cancer. Among patients alive in 2018 with colorectal cancer at age 55–64 years, 48% were already cured (had reached their specific TTC), while the cure prevalence (lifelong probability to be cured from cancer) was 89%. Cure prevalence became 97.5% (2.5% will die because of their neoplasm) for patients alive >5 years after diagnosis.

**Conclusions:**

This study represents an addition to the current knowledge on the topic providing a detailed description of available indicators of prevalence and cancer cure, highlighting the links among them, and illustrating their interpretation. Indicators may be relevant for patients and clinical practice; they are unambiguously defined, measurable, and reproducible in different countries where population-based cancer registries are active.

## 1 Introduction

Unlike other indicators of cancer burden (i.e., incidence, survival, or mortality), complete prevalence cannot be directly observed by cancer registries (CRs) because cancer survivors diagnosed before the start of registration are not included in the CR databases. The more recently the CR started registration, the greater the number of unobserved survivors (1). Therefore, complete prevalence and indicators of cancer cure, almost always based on statistical models, are reported less frequently than other indicators of cancer burden.

In the last decade, some epidemiologic investigations have explored the issue of estimating cancer cure in high-income countries (2–10), even if the usefulness to estimate indicators of cancer cure is held back by the lack of a shared definition of cure (11, 12). Nevertheless, several indicators of “cancer cure” have been proposed, particularly, the following: the *cure fraction* or the estimated probability of cure among incident cases (13, 14); the *time to cure*, the time necessary to make the excess risk of death due to cancer negligible (3, 4, 8, 10); *already cured* or the proportion of prevalent cases that have already reached the time to cure in a specific point in time (4); and *cure prevalence* or the proportion of all prevalent cases not expected to die due to their cancer (4, 15).

This article aimed to provide a complete and detailed description of the methodology and the procedures needed to derive complete prevalence and indicators of cancer cure from population-based CR data. The description has been accompanied by an application using the latest available Italian data. Improvement in the previously used algorithms (4, 5, 15, 16) to calculate cure indicators has been described, as well as validations of survival models and indicators. Finally, the epidemiological interpretation of indicators and the links among them are highlighted, with a discussion of assumptions made and their limitations.

## 2 Materials and methods

### 2.1 Study population

**Figure 1:**
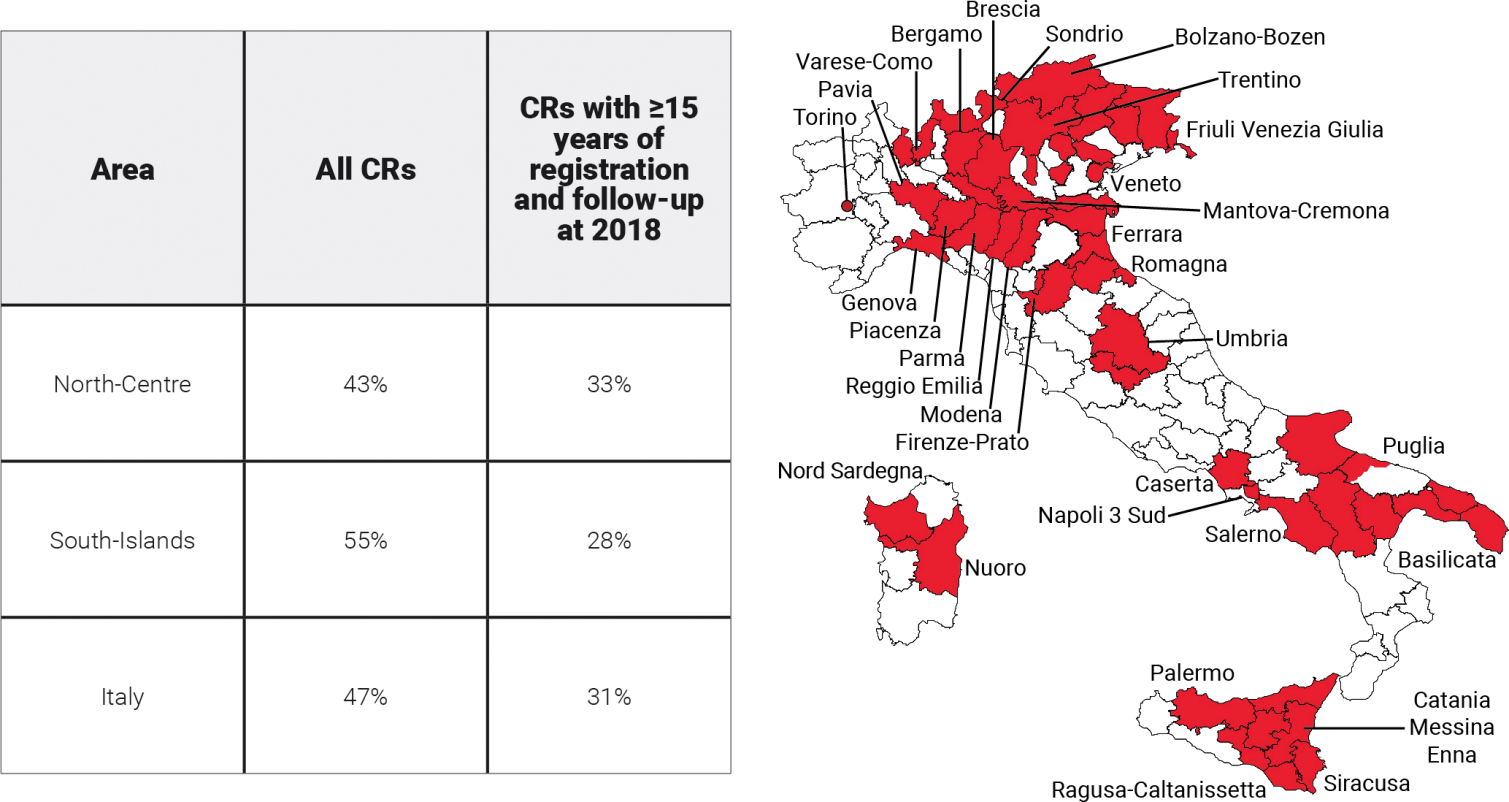
Areas and proportions of the Italian population included in the analyses. North-Centre includes Umbria and northern CRs.

This study included 31 population-based Italian CRs with at least 9 years of registration and patient vital status ascertainment at least 1 year after the last incidence date. By the end of 2017, the maximum duration of registration ranged from 9 to 40 years, with a median of 22 years (**Table 1**). Twenty CRs are located in north-central Italy [i.e., homogeneous areas in terms of incidence and survival (16)] and 11 in the South-Islands. CRs coverage varied with regards to the population size (0.2 to 2.8 million inhabitants), and overall, they cover more than 28 million people of all ages (43% of the population in north-central Italy, 55% in the South-Islands, and 47% overall; **Figure 1**). Since a key methodological point for the estimation of cure indicators is the availability of reliable estimates of “long-term” incidence and survival in the population of interest, Italian CRs with at least 15 years of registration (**Table 1**) and complete follow-up at the end of 2018 were included for the estimation of model-based incidence and survival. The geographical representativeness of these CRs is similar (~30%) between the north-central area and the South-Islands. Up to 1 January 2018, nearly 3.3 million (3,276,906, **Table 1**) incidents of malignant cancer cases were diagnosed in nearly 3 million (2,957,828) men and women, of all ages, in areas covered by CRs. They were two times higher than the number of cases included in the previous Italian report (17), including 443,901 female breast cancer cases, and 420,726 colorectal and 370,034 lung cancers (**Table 2**). For breast and colorectal cancer patients, prevalence and indicators of cancer cure were also calculated by stage at diagnosis including information from CRs with<33% of missing stage information for at least 15 consecutive years (i.e., respectively from six CRs for breast cancer and five CRs for colorectal cancer, approximately 6% of the Italian population) (**Table 1**).

**Table 1:** Period of registration, population, and incident cases in Italian cancer registries, 1978–2017.

Cancer registry	Period of registration	Years of registration	Population on 1 January 2018 (×1,000)	Incident cases up to 2017^a^
Basilicata	2005–2017	13	563	38,934
Bergamo	2007–2017	11	1,111	73,172
Bolzano-Bozen^b^	1995–2017	23	528	59,084
Brescia^b^	1999–2017	19	1,162	128,909
Caserta	2008–2016	9	916	38,830
Catania-Messina-Enna^b^	2003–2017	15	1,870	140,024
Ferrara^b^	1991–2017	27	348	72,436
Firenze-Prato (Florence)^b^	1985–2016	32	1,269	237,326
Friuli Venezia Giulia^b^	1995–2017	23	1,211	200,985
Genova (Genoa)^b, c^	1993–2016	24	836	158,893
Mantova-Cremona^b^	1999–2016	18	763	75,897
Modena^b^	1988–2017	30	703	121,185
Napoli 3 Sud (Naples)^b, c^	1996–2017	22	1,179	79,628
Nord Sardegna^b^	1992–2015	24	329	38,879
Nuoro	2003–2015	13	209	14,678
Palermo^b^	2003–2017	15	1,205	90,021
Parma^b, c^	1978–2017	40	450	104,062
Pavia^b^	2003–2017	15	546	55,825
Piacenza	2006–2017	12	287	24,565
Puglia (Apulia)	2006–2017	12	2,760	179,070
Ragusa-Caltanissetta^b^	1981–2017	37	588	56,429
Reggio Emilia^b, c^	1996–2017	22	534	66,768
Romagna^b, c^	1993–2017	25	1,126	179,599
Salerno^b^	1996–2017	22	1,091	102,970
Siracusa (Syracuse)^b^	1999–2017	19	401	35,072
Sondrio^b, c^	1998–2017	20	181	21,943
Torino (Turin)^b^	1985–2015	31	861	171,960
Trento^b^	1995–2017	23	540	65,203
Umbria^b^	1994–2017	24	885	120,371
Varese-Como	1990–2015	26	885	124,192
Veneto^b^	1990–2017	28	2,122	355,631
				
All CRs			28,057	3,276,906
				
Italy			59,937	
				

a Malignant cancer except non-melanoma skin cancer (ICD-10: C00–C43, C45–C66, C68–C96) and bladder cancer (C67, D09.0, D30.3, D41.4).

b CRs with at least 15 years of incidence are included to estimate the completeness index (using model-based incidence and survival, 47% of all incident cases).

c CRs with information on the stage of colorectal and breast cancer (Reggio Emilia CR only for breast cancer).

**Table 2:** Cancer sites or types and number of cases included: Italian cancer registries, 1978–2017.

Site or type	ICD-10	ICD-O-3 (T and M) or TNM	Cases^a^
All malignant cancers but the skin	C00–43, C45–96, D09.0, D30.3, D41.4		3,276,906
Head and neck	C00–14, C30–32		115,794
Oral cavity	C01–14		60,917
Mouth (excluded Base of Tongue)	C02–06		26,870
Salivary glands	C07–08		7,012
Oropharynx	C01, C09–10		13,314
Nasopharynx	C11		4,933
Esophagus	C15		22,916
Stomach	C16		153,726
Small intestine	C17		10,203
Colorectal	C18–C21		420,726
Colorectal, Stage I^b^	C18–C21	Stages I	7,874
Colorectal, Stage II^b^	C18–C21	Stages II	12,229
Colorectal, Stages III–IV^b^	C18–C21	Stages III–IV	18,989
Colon	C18		291,678
Rectal	C19–20		119,832
Anus	C21		9,216
Liver	C22		110,888
Hepatocellular carcinoma		C22.0–C22.1, 8170–8175, 8970	48,964
Intrahepatic cholangiocarcinoma		C22.0–C22.1, 8013, 8020, 8041, 8154, 8160–8162, 8180, 8240, 8246, 8249, 8470	6,905
Other hepatic cancer		C22.0–C22.1, any morphology except: 8013, 8020, 8041, 8154, 8160–8162, 8170–8175, 8180, 8240, 8246, 8249, 8470, 8970, 8800–8991, 9020, 9040–9044, 9050–9055, 9120–9133, 9140, 9150, 9170, 9180, 9220, 9231, 9240, 9251, 9260, 9364–9365, 9473, 9540, 9560–9571, 9580–9581, 9590–9989	55,137
Gallbladder	C23–24		43,842
Pancreas	C25		103,073
Larynx	C32		43,956
Lung bronchus trachea	C33–34		370,034
Bone	C40–41		6,453
Skin melanoma	C43		86,824
Mesothelioma	C45		13,659
Kaposi sarcoma	C46		8,007
Connective tissue	C47, C49		17,580
Soft tissue sarcoma^c^		All cancers sites except C40.0–C41.9, C32.3; C33.9; C34.0; C30.0; C30.1 (includes unknown primary sites): 8710, 8711, 8714, 8800, 8801, 8802, 8803, 8804, 8805, 8806, 8810, 8811, 8812, 8813, 8814, 8815, 8825, 8830, 8832, 8833, 8840, 8842, 8850, 8851, 8852, 8853, 8854, 8855, 8857, 8858, 8890, 8891, 8894, 8895, 8896, 8900, 8901, 8902, 8910, 8912, 8920, 8921, 8930, 8931, 8933, 8934, 8935, 8959, 8963, 8964, 8990, 8991, 9020, 9040, 9041, 9042, 9043, 9044, 9120, 9124, 9130, 9133, 9137, 9150, 9170, 9180, 9181, 9182, 9183, 9185, 9186, 9187, 9192, 9193, 9194, 9195, 9220, 9231, 9240, 9251, 9252, 9260, 9364, 9365, 9540, 9542, 9560, 9561, 9571, 9580, 9581 All cancer sites except C7–C8; C40.0–C41.9; C32.3; C33.9; C34.0; C30.0; C30.1; C60; C44; C63.2: 8940 C49 only: 8004 All cancer sites except C40.0–C41.9, C32.3; C33.9; C34.0; C30.0; C30.1, C56, C71, C72: 9473 All cancer sites except C40.0–C41.9, C32.3; C33.9; C34.0; C30.0; C30.1, C71, C72: 9503	33,054
Bone sarcoma^c^		C40.0–C41.9, C32.3; C33.9; C34.0; C30.0; C30.1: 8800, 8801, 8802, 8803, 8804, 8805, 8806, 8810, 8811, 8812, 8815, 8830, 8840, 8850, 8851, 8852, 8853, 8854, 8855, 8890, 8891, 8894, 8895, 8896, 8900,8901, 8902, 8910, 8912, 8920, 9040, 9041, 9042, 9043, 9044, 9120, 9124, 9130, 9133, 9150, 9170, 9180, 9181, 9182, 9183, 9184, 9185, 9186, 9187, 9192, 9193, 9194, 9195, 9220, 9221, 9230, 9231, 9240, 9242, 9243, 9250, 9260, 9364, 9473, 9540, 9560, 9561, 9571, 9580, 9581 Only in C40.0–41.9: 8004 All cancer sites: 9370, 9371, 9372	5,127
GIST^c^		8936	3,482
Breast (women only)	C50		443,901
Breast, Stage I^d^	C50	Stage I	25,050
Breast, Stage II^d^	C50	Stage II	18,493
Breast, Stages III–IV^d^	C50	Stages III–IV	8,568
Vagina and vulva	C51–52		12,789
Vulvar SCC		C51.0–C51.9, 8051–8084	8,073
Cervix uteri	C53		25,402
Corpus uteri	C54		72,447
Ovary	C56		48,830
Penis	C60		4,124
Prostate	C61		318,705
Testis	C62		17,646
Kidney	C64–66, C68		106,219
Bladder	C67, D09.0, D30.3, D41.4		236,967
Brain and CNS	C70–72		51,609
Thyroid	C73		82,532
Thyroid, papillary	C73	8050, 8052, 8260, 8263, 8340–8344, 8350, 8450	65,989
Thyroid, follicular	C73	8290, 8330–8335	6,753
Thyroid, anaplastic	C73	8012, 8020–8035, 8190, 8337	1,456
Thyroid, medullary	C73	8246, 8345–8347, 8510	2,626
Hodgkin lymphoma		9650–9667	20,107
Non-Hodgkin lymphoma	C82–85, C96		112,808
CLL/SLL		9670, 9823	33,834
NHL, DLBC		9678–9684	35,040
NHL follicular		9675, 9690–9698	17,838
Myeloma (plasma cell)		9731–9734	47,029
Leukemia	C91–95		82,873
Precursor cell acute lymphoblastic leukemia		9727–9729, 9835–9837	8,121
Acute myeloid leukemia		9840, 9861, 9866–9867, 9870–9874, 9891–9931	25,516
Chronic myeloid leukemia		9863, 9875	8,960

a For the combinations of cancer types, only the first primary tumor was included.

b CRs of Genova, Sondrio, Parma, Romagna, and Napoli 3 Sud (5.3% of the Italian population), 1997–2017.

c RARECARE (18).

d CRs of Genova, Sondrio, Parma, Reggio Emilia, Romagna, and Napoli 3 Sud (6.1% of the Italian population), 1997–2017.

### 2.2 Cases and groupings

Prevalence and indicators of cancer cure were calculated for all malignant cancers and 62 types or their combinations (**Table 2**) using ICD-10 classification. In addition, ICD-O-3 topography and morphology codes were used to define specific subtypes (18). Urinary bladder cancers with benign or uncertain behavior and *in situ* tumors were also accounted for (ICD-10: D09.0, D30.3, D41.4), while non-melanoma skin cancers (ICD-10: C44) were excluded. To estimate cancer-specific prevalence for each patient, we considered only the first primary cancer occurring in that specific site. Multiple primary cancers in different organs diagnosed in the same person were included in each site-specific analysis. For the combinations of cancer types, only the first primary tumor was considered.

### 2.3 Quality checks

To ensure comparability and to verify the completeness of CR incidence and follow-up data and in agreement with well-established international guidelines and standards (16, 19), the following three quality indicators were calculated for each CR: the proportion of cases known by death certificate only (DCO), a common indicator for cancer registration accuracy and completeness; the proportion of microscopic verifications (MVs), an indicator of the quality of the documentation available to the registry; and the percentage of cases lost to follow-up before 5 years (<5% loss leads to little bias in survival analyses) (20).

### 2.4 Limited duration prevalence

Limited duration prevalence (LDP) on 1 January 2018 (i.e., index date) was computed from observed incidence and follow-up data for each CR. LDP includes only cases diagnosed after the start of the CR activity and was calculated up to the maximum registration period (between 9 and 40 years), stratified by cancer type, sex, 5-year age groups (from 0–4 to 80–84, and 85+), and years since diagnosis. The calculations were performed by counting the number of persons known to be alive at the index date and adjusting for those lost to follow-up, as implemented in the SEER*Stat software (21). For the eight CRs with the last year of incidence before 2017 (i.e., 2015 or 2016), LDP was calculated for the last 3 years available and projected to 1 January 2018 by CRs, cancer type, sex, age, and time since diagnosis, using a linear regression model with the calendar year as an independent variable (17, 22).

### 2.5 Survival

Reliable estimates of long-term (>15 years) survival are crucial for both the estimation of cure indicators and the complete prevalence through statistical modeling and completeness index estimation (see below). They should be representative of the population under study and sufficiently robust to allow modelization of survival in the distant past or near future.

Net survival (NS) is the probability that cancer patients survive their cancer up to a given time since diagnosis, after controlling for competing causes of death. NS allows comparison of populations as if the disease under study was the only possible cause of death. NS was calculated for cases of all ages diagnosed in 1991–2017 and follow‐up until the end of 2018, using the cohort method and the Pohar Perme approach (23), as implemented by the SEER*Stat software (21).

DCO only and cases incidentally diagnosed at autopsy were excluded from the analysis.

Expected survival was computed from the regional life tables provided by the Italian National Institute of Statistics for each CR area, stratified by age (in years), sex, and calendar year (24).

For the pool of CRs with ≥15 years of incidence (**Table 1**) and follow-up until 2018, NS estimation was calculated by cancer type, sex, age at diagnosis (0–44, 45–54, 55–64, 65–74, 75+ years), and period of diagnosis (in 3-year periods from 1991–1993 to 2015–2017). For cancers with available stage information (i.e., breast and colorectal), NS estimation was calculated in the period 1997–2017 for a subset of CRs.

Conditional net survival (CNS) was calculated as the probability of surviving an additional number of years, given that patients already survived *t* years (16).

Model-based net survival was calculated using mixture cure models which consider a population as a mixture of two groups: the cured (i.e., patients who will have the same life expectancy as the general population) and not cured (i.e., the patients expected to die due to their cancer) (13). Consequently, the mixture cure model is a combination of two models which estimate both the proportion of cured patients (i.e., CF: the cure fraction) and the survival function of the remaining “not-cured” patients (i.e., fatal cases, 1 − *CF*).

For any cancer type and sex, the model which best fit NS and CNS was explored starting from an age-stratified Weibull model. When this model did not converge, alternative models were explored, i.e., Weibull without age stratification, age-stratified exponential, or exponential without age stratification. For rare cancer types, with few patients in some strata of sex or age, parameters were calculated by collapsing the relevant strata as specified in **Supplementary Table 1**. Parameters were estimated using the SAS NLIN procedure. The goodness of fit of “model-based” NS to “observed” NS was evaluated by likelihood ratio tests and by visual comparison (4, 25, 26), for each cancer type, period of diagnosis, sex, and age group.

### 2.6 Incidence

Incidence function is needed to describe the risk of being diagnosed with cancer, throughout the life span of each birth cohort in the population (i.e., to estimate the incidence before the start of registration by CRs and completeness index, see below). In the present study, a sixth-degree polynomial on age was the best-fitting model and was used to estimate incidence rates by cancer type and sex (27).

Age and cohort parameters of the incidence function were estimated using SAS logistic procedure by fitting crude incidence rates of patients diagnosed between 1990 and 2014 (in 5-year periods) in the same CRs used for survival modelization, between 1995 and 2014 for breast and colorectal cancers by stage. Incidence data were categorized according to cancer type, sex, 5-year age groups, and birth cohort (<1899, 1900–1904, …, 2000–2014). The goodness of fit of the incidence models was assessed by the Akaike information criterion (AIC) as well as by visual comparison between estimated and observed rates.

### 2.7 Completeness index

The completeness index (*R_L_*) represents the proportion of prevalence observed from CRs with *L* years of registration, and it is necessary to calculate the complete prevalence as *LDP/R_L_* (28, 29). *R_L_* represents the percentage of completeness of LDP and varied between 0 and 1, depending on the prevalence observed by the registry. Values close to 1 indicate a high level of completeness and, therefore, a small correction to be applied to the observed prevalence. *R_L_* was calculated by cancer type and sex, using the model-based net survival (*NS*) and incidence (*I*):


$$R_{L}(x)=\frac{\sum_{t=x-L}^{x}I\left(t\right)NS\left(t,x-t\right)}{\sum_{t=0}^{x}I\left(t\right)NS\left(t,x-t\right)}$$

where *x* is the age at prevalence and *x − t* is the age at diagnosis. The completeness index was calculated using the ComPrev software (30).

To evaluate the effect of using different periods of incidence and survival on the completeness index estimates and complete prevalence, a validation was conducted using two registries with a long observation period: Veneto (28 years of duration, in the north, with high prevalence and relatively high incidence rate in comparison with all of Italy) and Ragusa (37 years, in the south, a low incidence and prevalence area). We compared the maximum observed LDP for the two CRs (*LDP_max_* at 28 years for Veneto CR and at 37 years for Ragusa CR) with the LDP of the same duration ( $\hat{LDP_{max}}$ ) estimated by completing LDP at 15 years using three different completeness indexes *R_L_* (*x*): one based on the 1990–2017 incidence and survival, one on the 2003–2017, and using the *R_L_* (*x*) provided by the ComPrev software, estimated on SEER data. The calculation has been done as


$$\hat{LDP_{max}}=\;\frac{LDP_{15}}{R_{15}}\cdot R_{max}$$

where *R_max_* is the index at 28 years for Veneto CR and at 37 years for Ragusa CR.

### 2.8 Complete prevalence in 2018

Complete prevalence (Prev) was calculated on 1 January 2018. Estimation was based on observed LDP and, for the period before the start of registration, on the estimated fraction of prevalence not observed in the recorded data (28, 29). The estimated complete prevalence at age *x* (*Prev(x)*) includes all incident cases diagnosed at any age and can be split into two components, observed LDP (durations from *x − L* to *x* years) and estimated unobserved ones (from *0* to *x − L − 1*):


$$Prev\left(x\right)=LDP_{L}(x)+Prev_{L}^{unobs}(x)=\frac{LDP_{L}\left(x\right)}{R_{L}\left(x\right)}$$

*Prev(x)* was calculated as absolute numbers and proportions by CR, cancer type, sex, and age at prevalence.

For each registry with *L*<40 years, we also estimated the annual LDP up to 40 years after diagnosis:


with *d=L+1, …40*
$$\;LDP_{d}\left(x\right)=LDP_{L}\left(x\right)\cdot \frac{\;R_{\;d}\left(x\right)}{\;R_{\;L}\left(x\right)}$$

This estimation by years since diagnosis will be used for the calculation of *already cured* patients described in Section 2.11.

The absolute number of prevalent cases in Italy was obtained as the sum of proportions of prevalence estimates (age-, sex-, and cancer type-specific, obtained pooling CRs in the north-central area and in the South-Islands included in this study) multiplied by the corresponding Italian population in the same areas at the index date (24).

### 2.9 Complete prevalence projections

To obtain complete prevalence projections after 2018 for all CRs, and up to 2018 for CRs with missing incidence data in 2016 or 2017, the complete prevalence was estimated over the last three calendar years available by CR, cancer type, sex, and age. The number of prevalent cases was projected using a linear regression model with the calendar year as an independent variable, assuming that prevalence would follow a linear function. This simplified assumption (linear and constant trend) may not be valid for long-term projections, but it is reasonable in the medium-term (e.g., 10 years) (17) for common cancer types. The proportions of prevalence estimates (age-, sex-, and cancer type-specific) from CRs in the north-central area and the South-Islands included in this study were multiplied by the corresponding Italian population in the same area at the index date by sex and age (24). It should be noted that the Italian population is observed until 2021 and forecasted in subsequent years when we used estimates based on the “median” forecast scenario.

### 2.10 Life expectancy of fatal cases, cure fraction, and time to cure

**Figure 2:**
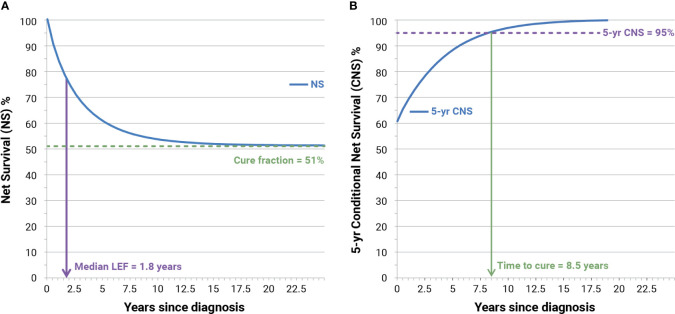
Examples of calculation of cure fraction, median life expectancy of fatal (LEF) cases **(A)**, and time to cure **(B)** for Italian patients (men and women) with colorectal cancer diagnosed in 1995 at age 55–64 years. NS, model-based net survival; CNS, conditioned NS.

Life expectancy of fatal (LEF) cases is the survival experienced by the 50th percentile (i.e., median LEF) of fatal cases. In the example (**Figure 2A**) LEF was 1.8 years corresponding to NS = 75.7% half of those above the green dashed line. Not all cancer patients die because of their neoplasm and, for most cancer types, the NS curve reaches a plateau after a certain number of years (approximately 15 years). Notably, we can observe that a small or large proportion of patients will not die because of their neoplasm even if the plateau is not reached.

The CF represents the proportion of incident patients who experience, at diagnosis, the same life expectancy (mortality rates) as their peers in the general population (51%, **Figure 2A**). CFs have been calculated from mixture model-based NS and represent asymptotical values of NS when the time since diagnosis increases toward “infinity.” Since the life expectancy of people with or without cancer is less than asymptotical, and to highlight connections and differences between CF and long-term NS, we also calculated NS at 50 years after diagnosis, at attained ages 90 and 100 years.

CF for all patients was calculated as a weighted average of age-specific CF, each weight being the proportion of incident cases in the corresponding age group. Changes in CF over time were estimated by using the *period* parameter of the survival function, which represents the effects of the “year of diagnosis” and can be modified assuming a linear effect of the period of diagnosis.

**Figure 2B** shows also the increase of 5-year CNS (blue curve) according to time since diagnosis. When 5-year CNS approaches 100%, patients reach the same life expectancy (mortality rates) as that observed in the general population who is free from cancer. The assumption is that time to cure (TTC) is reached when 5-year CNS becomes higher than 95% (3), thus assuming the residual 5% excess mortality to be clinically negligible. In the example (**Figure 2B**), the TTC is reached after 8.5 years.

### 2.11 Cure prevalence and already cured

Cure prevalence (CurePrev) is defined as the proportion of prevalent cancer patients who will not die as a result of cancer. This indicator was estimated by


$$CurePrev_{t}(x)=\frac{CF_{x-t\;}*\;Prev_{t\;}\left(x\right)}{\left[NS_{x-t}\left(t\right)+NS_{x-t}\left(t-1\right)\right]/2}$$

where *CF_x_*_−_ *_t_* and *NS_x_*_−_ *_t_ (t)* are, respectively, the cure fraction and the net survival of patients diagnosed at age *x* − *t* and follow-up time *t*, to obtain *CurePrev_t_* (*x*), the cure prevalence at attained age *x.* In the present study, the mean NS at the beginning and the end of the year has been applied to each year since diagnosis. In other words, this indicator was computed as the number (or proportion) of prevalent cases having the same life expectancy (mortality rates) as the corresponding group (i.e., same sexes and age) in the general population, conditioned to be alive *t* years after diagnosis. For each cancer type and sex, the overall *CurePrev* was calculated as


$$CurePrev=\;\frac{\sum_{x}^{\;}\left(\sum_{t}CurePrev_{t}\left(x\right)\right)}{Prev_{TOT}}$$

summing up estimates over all ages at prevalence *(x)* where duration is up to the maximum 40 years after diagnosis and *Prev_TOT_* is the overall complete prevalence for all age groups considered.

**Figure 3:**
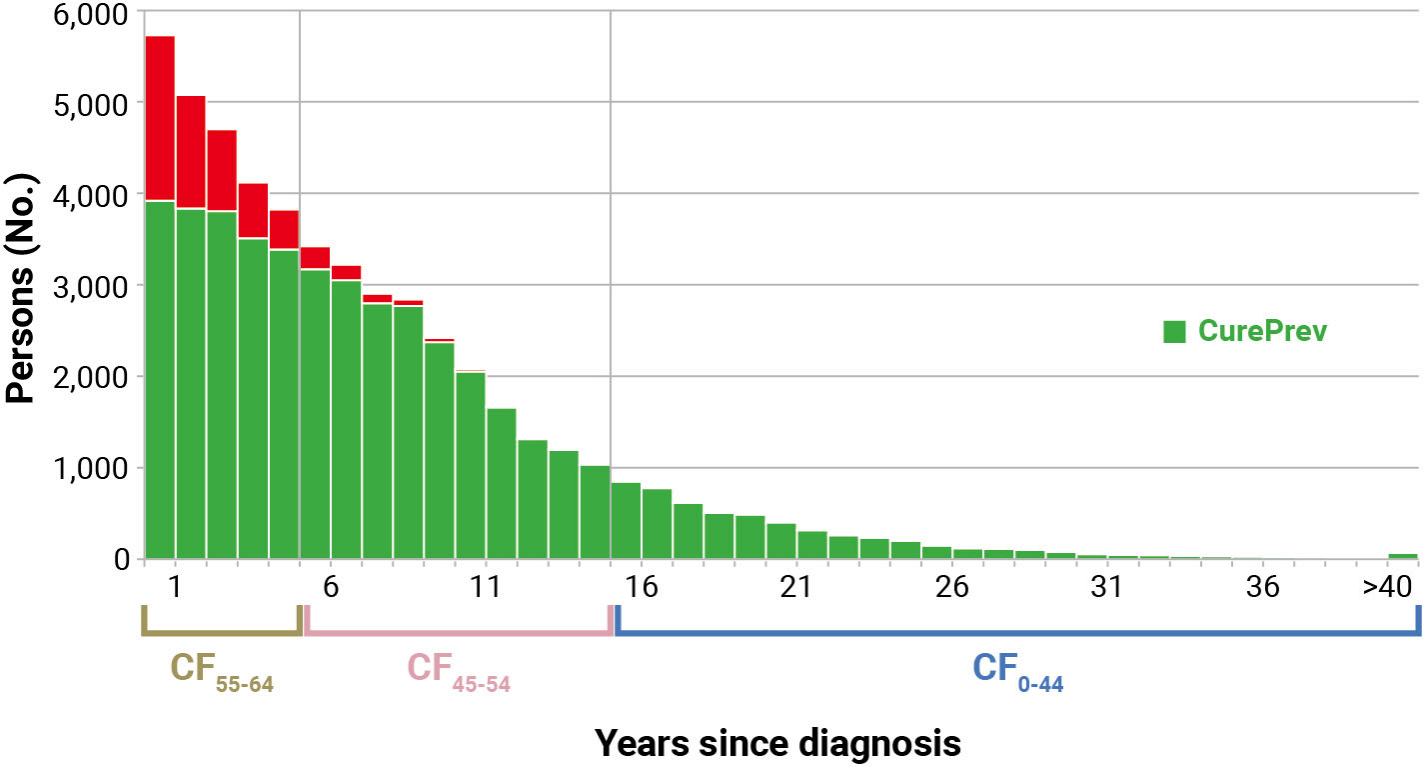
Calculation of cure prevalence (CurePrev) for Italian colorectal cancer patients (men and women), aged 55–64 years who were alive in 2018 (January 1st). Calculated applying to complete prevalence at attained age 55–64 the cure fraction (CF) calculated for age at diagnosis, according to years since diagnosis (Section 2.11). The red part of each bar includes cases who are expected to die because of their cancer.

**Figure 3** shows an example of the calculation of *CurePrev* in which each annual vertical bar represents the number of patients alive *n* years after diagnosis. The green part of each bar includes cases having the same life expectancy as their peers in the general population (i.e., CF for those alive at that point) and markedly increases with time since diagnosis. Conversely, the red part of each bar includes cases who are expected to die because of their cancer and decreases with time since diagnosis.

To the same distribution of prevalent patients presented in **Figure 3**, TTC can be applied. Consequently, already cured (*Prev*(>*TTC*)) is defined as the proportion of patients who already reached TTC, defined here as 5-year CNS >95%. It was calculated as the sum of prevalent patients by more than TTC


$$Prev\left(>TTC\right)=\;\frac{\sum_{x}^{\;}\sum_{t>TTC}Prev_{t\;}\left(x\right)}{Prev_{TOT}}$$

**Figure 4:**
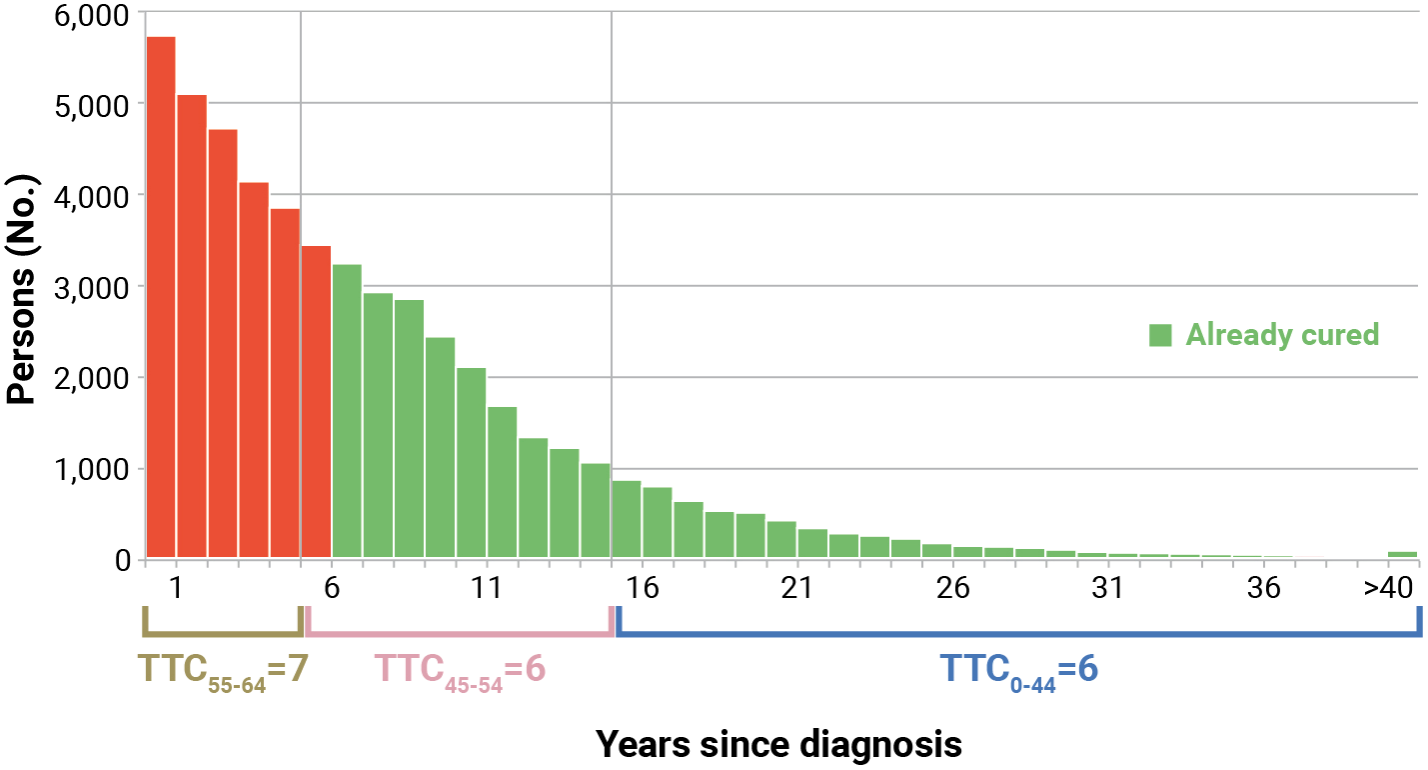
Calculation of already cured (Prev>TTC) for Italian colorectal cancer patients (men and women), aged 55–64 years who were alive in 2018 (January 1st). Calculated applying to complete prevalence at attained age 55–64 the time to cure (TTC) calculated for age at diagnosis, according to years since diagnosis (Section 2.11). The red part includes patients who have not yet reached TTC.

Estimates of *TTC* were calculated using age at diagnosis of patients, while *Prev_t_* was based on the age of prevalent cases. To overcome this discrepancy, we applied the TTC estimated at different ages at diagnosis to the distribution of prevalent cases at the attained age. In the example (**Figure 4**), prevalent patients at the attained age of 55–64 years (median 60 years) alive in 2017 had a TTC = 7 years (first 5 years) if diagnosed in the same age group, while they had TTC = 6 years if they were diagnosed at age 45–54 years (median 50 years). Consequently, patients prevalent at 60 years of age who were diagnosed at the same age can be considered cured after 7 years (not yet reached) and after 6 years if diagnosed younger. Therefore, among these groups, those alive >6 years after diagnosis were considered already cured. The green part of **Figure 4** includes already cured patients, while the red part includes those who have not yet reached TTC.

CurePrev included both patients surviving a shorter period than TTC (they will reach it in the future) and a small proportion (<5%, by definition) of already cured (*Prev*(>*TTC*)) with a small excess risk of death, in comparison with their peers in the general population. Notably, only *Prev*(>*TTC*) patients can be individually identified.

In **Supplementary Figure 1**, the steps needed to calculate complete prevalence on 1 January 2018, projections for the following years, and indicators of cancer cure are summarized. The links among the indicators are also shown and which of them are preliminary to the estimation of the others. For instance, survival estimates are sufficient to calculate CF and TTC. Incidence estimates are also necessary for the calculation of the completeness index and, thus, the complete prevalence. Finally, both estimates of complete prevalence per year after diagnosis and estimates of TTC are needed to calculate the number of already cured patients.

### 2.12 Ethical approval

The Italian legislation identifies regional health authorities as collectors of personal data for surveillance purposes without explicit individual consent. The approval of a research ethics committee was not required, since this study is a descriptive analysis of pseudonymized cancer data collected by the registries, without any direct or indirect intervention on patients (31).

## 3 Results

### 3.1 Quality checks

Three major indicators of data completeness and quality of Italian CRs are shown in **Table 3**. In the last 10 years of registration (i.e., 2008–2017), the overall percentage of microscopically verified cases was 86.3% with only one CR<80%. The proportion of cases known by death certificate only or with an unknown base of diagnosis was 1.1% with only one CR with a proportion >2%. The percentage of cases lost to follow-up before 5 years was 0.6%, with only 7 out of 31 CRs >1%.

**Table 3:** Quality indicators by cancer registry for cases^a^ diagnosed in 2008–2017.

Cancer registry	Records (*n*)	Microscopic verifications (%)	DCO—unknown (%)	Lost to follow-up<5 years (%)
Basilicata	30,501	78.6	0.7	0.1
Bergamo	66,634	89.1	1.1	0.4
Bolzano-Bozen	27,819	91.0	0.9	0.0
Brescia	70,240	80.4	1.6	0.4
Caserta	38,830	86.5	1.6	0.3
Catania-Messina-Enna	97,131	87.4	1.6	1.4
Ferrara	28,756	84.9	0.4	0.9
Firenze-Prato	75,628	83.9	1.0	0.9
Friuli Venezia Giulia	89,495	89.9	0.4	0.5
Genova	58,776	84.6	1.0	0.2
Mantova-Cremona	46,150	86.3	0.7	0.2
Modena	46,445	87.8	0.6	0.0
Napoli 3 Sud	56,903	86.4	1.3	0.2
Nord Sardegna	15,003	84.9	1.7	0.1
Nuoro	9,476	82.8	1.7	0.1
Palermo	62,417	83.2	3.4	0.1
Parma	31,273	88.1	0.7	1.7
Pavia	37,100	82.5	0.9	0.5
Piacenza	20,186	81.2	1.2	1.6
Puglia	151,272	84.5	1.2	0.8
Ragusa-Caltanissetta	27,749	83.1	1.9	0.9
Reggio Emilia	32,221	90.1	0.2	1.1
Romagna	79,120	88.1	0.7	0.1
Salerno	51,616	83.2	1.4	2.5
Siracusa	19,877	82.9	1.8	1.3
Sondrio	11,050	83.7	2.0	0.0
Torino	48,933	88.8	1.6	1.4
Trentino	31,642	87.5	0.3	0.8
Umbria	54,518	92.9	0.4	0.6
Varese-Como	69,044	89.7	0.8	0.4
Veneto	138,489	87.9	0.8	0.4
				
All CRS	1,624,294	86.3	1.1	0.6

DCO, death certificate only.

a Malignant cancer except non-melanoma skin cancer (ICD-10: C00–C43, C45–C66, C68–C96) and bladder cancer (C67, D09.0, D30.3, D41.4).

### 3.2 Validation of survival models

**Figure 5:**
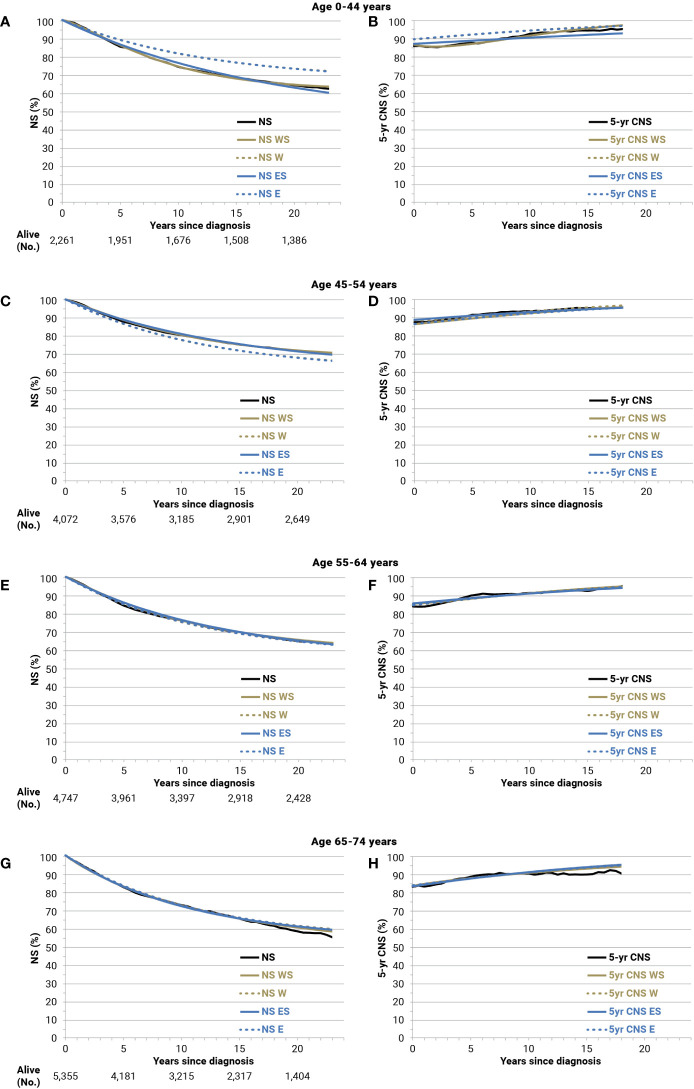
Net survival (NS), 5-year conditional NS (5-year CNS), and corresponding model-based estimates until 24 years of follow-up for breast cancer patients (all stages) diagnosed in 1994–1996 and followed up until 2018 by age group: Age 0-44 years **(A, B)**; 45-54 **(C, D)**; 55-64 **(E, F)**; 65-74 **(G, H)**. W, Weibull; WS, Weibull, age-stratified; E, exponential; ES, exponential, age-stratified.

The comparisons of NS and 5-year CNS with corresponding model-based curves were made for all cancer types and sex. As an example, results for the cohort of breast cancer patients diagnosed in 1994–1996 and followed up until 24 years after diagnosis are shown by age groups in **Figure 5**. Overall, these comparisons and those for the 3-year period cohorts, from 1991–1993 to 2015–2017 (not shown), suggested a very good fit, not only for age-stratified Weibull models but also for exponential models, to estimate long-term model-based survival and cure indicators for breast cancer patients. In particular, for the 2,261 women with breast cancer at age 0–44, the 20-year NS was 64.4% and overlapping values emerged for the age-stratified Weibull models (NS WS = 64.7%) (**Figure 5A**, solid gold line). Some differences emerged for the age-stratified exponential models (NS ES = 63.0%) (solid blue line), broader for Weibull or exponential models without age stratification (dashed lines: 73.5% and 73.4%, respectively). The corresponding observed 5-year CNS 15 years after diagnosis was 93.9% (**Figure 5B**), slightly below the threshold for TTC (i.e., 95%), while they were 95.1% when calculated by the age-stratified Weibull or exponential models, 95.6% for Weibull, and 95.8% for the exponential models without age stratification. For patients with breast cancer diagnosed at ages 45–54 years (4,072 women) or 55–64 years (4,747 women), negligible differences emerged between observed and estimations of NS or 5-year CNS based on the age-stratified models (Weibull or exponential) (**Figures 5C–F**). The same applies at ages 65–74 years (5,355 women) at least until 15 years after diagnosis or attained at the age of 80–89 years (**Figures 5G, H**). The results of the observed and best-fitting model-based NS and 5-year CNS are also presented for patients with breast cancer by stage at diagnosis (**Supplementary Figure 2**) and for patients with colorectal (**Supplementary Figure 3**) or prostate cancers and soft tissue sarcomas (**Supplementary Figure 4**). A good fit emerged for all of them.

**Supplementary Table 1** lists the survival model with the best fit by cancer type with appropriate adjustments for sex and age, if necessary.

### 3.3 Validation of incidence models

The comparisons between observed and model-based age-specific incidence rates are shown in **Supplementary Figure 5**. For all cancer types combined by sex, as well as for prostate and breast cancers diagnosed in the period 1990–2014, a very good fit emerged for incidence models to be included in the completeness index estimation. The same validations have been done for all cancer types, by sex and period.

### 3.4 Validation of the completeness index

In **Table 4**, frequent cancer types with relatively good prognoses (colorectal, breast, and thyroid cancers and skin melanoma) have been selected as examples in registries with relatively high (Veneto) or low (Ragusa) incidence rates. A less marked difference is expected for patients with poor prognosis or cancer types more frequently diagnosed at older ages when the proportion of patients living >15 years after diagnosis is low regardless. Differences<2% emerged for the four cancer types examined in the Veneto registry between the observed 28-year LDP and the same duration prevalence estimated starting from 15-year LDP using the completeness index calculated from Italian registries with a long-term period of incidence and survival (i.e., 1990–2018). Differences were more marked (+6.1% for colorectal cancer in men, +23.5% for thyroid in women) using only the completeness index based on shorter periods of incidence and survival (2003–2018) and also using the completeness index calculated on SEER data and provided with the ComPrev software (+3.5% for melanoma in men and +9.2% for thyroid in women). In addition, a consistent overestimation emerged for the 37-year LDP completed by the 15-year LDP for the Ragusa registry, approximately +5% using the completeness index based on Italian data 1990–2018 but greater than 10% for some cancer types using both the completeness index based on short period or SEER data (**Table 4**).

**Table 4:** Difference between the maximum duration prevalence calculated from 15-year limited duration prevalence (LDP), using different completeness indexes (*R_L_* (*x*))^a^, and observed maximum LDP for selected cancer types.

Registry (maximum duration) Cancer type	Sex	Max LDP			
		Observed	Calculated (%)^b^, using *R_L_* (*x*) from		
			Italy 1990–2018	Italy 2003–2018	SEER 1975–2005
Veneto (28 years)					
Colorectal	Men	8,184	8,143 (−0.5%)	8,680 (+6.1%)	8,196 (+0.2%)
Skin melanoma	Men	3,725	3,728 (+0.1%)	3,627 (−2.6%)	3,857 (+3.5%)
Breast	Women	30,792	31,135 (+1.1%)	32,004 (+3.9%)	30,115 (−2.2%)
Thyroid	Women	4,643	4,555 (−1.9%)	5,334 (+23.5%)	5,070 (+9.2%)
Ragusa (37 years)					
Colorectal	Men	886	931 (+5.1%)	1,004 (+13.3%)	949 (+7.1%)
Skin melanoma	Men	264	282 (+6.7%)	271 (+2.6%)	294 (+11.3%)
Breast	Women	2,763	2,947 (+6.7%)	3,089 (+11.8%)	2,849 (+3.1.%)
Thyroid	Women	698	723 (+3.5%)	895 (+28.1%)	858 (+22.8%)

a Calculated as described in Section 3.4.

b % represents the difference between calculated and observed.

### 3.5 Completeness index: comparisons

Values of *R_L_* (i.e., completeness index for different lengths of observation) are presented in **Table 5** for breast, colorectal, and prostate cancers and all cancer types. The *R_L_* increases with lengths of follow-up and with decreasing age. For colorectal cancer, *R*_20_ (i.e., for a 20-year duration) decreased from 97.2% at age 40–44 in men (96.0% in women) to 78.9% (75.0% in women) at 85+ years. *R*_30_ was approximately 100% until age 70 years and 10% higher than *R*_20_ for ages 70 years or more, while *R*_40_ was always above 98%. Values near 100% for a 20-year duration emerged for prostate cancers mainly diagnosed in older adults, while *R*_20_<80% was estimated for breast cancer patients aged >70 years (61.6% for 85+ years). In other words, in CRs with a 20-year duration, the LDP underestimated complete prevalence, with a loss of >20% for women with a previous cancer diagnosis aged 70 years or more (>10% in men) (**Table 5**).

**Table 5:** Completeness index (*R_L_*, %)^a^ by sex, age, length (*L*) of the observation period, and cancer type^b^.

Age groups (years)	Cancer type, sex							
	Colorectal cancer, men			Colorectal cancer, women				
	*L* = 20	*L* = 30	*L* = 40	*L* = 20		*L* = 30		*L* = 40
40–44	97.2	99.9	100.0	96.0		99.5		100.0
45–49	96.6	99.7	100.0	96.1		99.3		100.0
50–54	96.4	99.4	100.0	96.0		99.2		99.9
55–59	96.1	99.3	99.9	95.4		99.1		99.8
60–64	95.4	99.1	99.9	94.0		98.8		99.8
65–69	93.9	98.8	99.8	91.8		98.4		99.7
70–74	91.6	98.3	99.7	88.6		97.6		99.6
75–79	88.2	97.4	99.5	84.5		96.3		99.3
80–84	83.8	95.9	99.2	79.5		94.3		98.8
85+	78.9	93.7	98.6	75.0		91.7		98.1
	Prostate cancer, men			Breast cancer, women				
	*L* = 20	*L* = 30	*L* = 40	*L* = 20		*L* = 30		*L* = 40
40–44	99.0	99.0	99.6	99.8		100.0		100.0
45–49	100.0	100.0	100.0	99.3		100.0		100.0
50–54	100.0	100.0	100.0	98.0		99.9		100.0
55–59	100.0	100.0	100.0	95.0		99.8		100.0
60–64	99.9	100.0	100.0	90.2		99.2		100.0
65–69	99.8	100.0	100.0	83.9		97.7		99.9
70–74	99.3	100.0	100.0	77.1		95.0		99.6
75–79	98.0	100.0	100.0	71.2		91.1		98.8
80–84	94.9	99.9	100.0	66.0		86.1		97.2
85+	89.4	99.8	100.0	61.6		81.6		94.7
	All cancers, men			All cancers, women				
	*L* = 20	*L* = 30	*L* = 40	*L* = 20		*L* = 30		*L* = 40
40–44	81.7	91.9	98.9	91.0		96.2		99.3
45–49	83.4	92.1	97.3	91.5		96.8		98.7
50–54	85.1	93.2	96.8	90.9		97.1		98.7
55–59	86.8	94.4	97.1	89.1		97.0		98.8
60–64	88.2	95.4	97.8	86.3		96.4		98.8
65–69	89.4	96.1	98.3	82.7		95.2		98.7
70–74	90.0	96.2	98.6	78.5		93.3		98.3
75–79	88.9	96.1	98.7	73.9		90.7		97.6
80–84	85.7	95.5	98.4	68.6		86.9		96.4
85+	80.9	94.5	98.2	64.2		83.1		94.6

a Completeness index calculated from Italian registries with a long-term period of incidence and survival (i.e., 1990–2018).

b The extended version is available upon request for the most frequent cancer types by the annual length of observation period from 7 to 40 years.

In **Table 6**, four estimates of the proportions of prevalent cases observed up to 20 years after diagnosis *R*_20_(*x*) have been compared: those according to estimates made in Italy for 2006 (27), 2010 (22), and 2018 (present estimates), as well as those estimated on SEER data (30).

**Table 6:** Comparison of different completeness indexes for 20 years of length of the observation period (*R*_20_, %) for all cancers combined by sex and age groups.

Age groups (years)	Men				Women			
	*R*_20_, %				*R*_20_, %			
	Italy at			USA^d^	Italy at			USA^d^
	2018^a^	2010^b^	2006^c^		2018^a^	2010^b^	2006^c^	
0–4	100.0	100.0	100.0	100.0	100.0	100.0	100.0	100.0
05–09	100.0	100.0	100.0	100.0	100.0	100.0	100.0	100.0
10–14	100.0	100.0	100.0	100.0	100.0	100.0	100.0	100.0
15–19	100.0	100.0	100.0	100.0	100.0	100.0	100.0	100.0
20–24	94.3	94. 6	94.0	92.3	92.7	91.9	89.5	91.5
25–29	85.5	86.1	85.7	85.4	85.9	85.3	85.9	84.3
30–34	81.7	83.0	83.3	85.1	87.1	87.4	83.7	85.4
35–39	80.6	83.4	84.0	85.5	89.4	90.4	86.5	87.2
40–44	81.7	85.8	86.5	85.9	91.0	92.6	89.8	88.2
45–49	83.4	88.2	89.3	86.6	91.5	93.4	91.7	88.2
50–54	85.1	89.8	91.7	87.9	90.9	93.1	92.3	87.4
55–59	86.8	91.1	93.4	89.6	89.1	91.8	91.8	85.9
60–64	88.2	91.7	94.4	91.1	86.3	89.5	90.3	83.9
65–69	89.4	92.4	94.8	92.0	82.7	86.7	88.1	81.4
70–74	90.0	93.0	94.5	91.7	78.5	83.3	85.3	78.5
75–79	88.9	92.4	93.7	90.2	73.9	79.7	82.5	75.1
80–84	85.7	90.9	92.2	87.1	68.6	76.1	80.0	71.4
85+	80.9	88.6	90.1	83.3	64.2	73.6	76.4	67.6

a Based on Italian incidence and survival trends in 2018.

b Based on Italian incidence and survival trends in 2010 (22).

c Based on Italian incidence and survival trends in 2006 (27).

d Based on the SEER incidence and survival trends in 2018, estimated from data in 2005 (Race: White) (30).

*R*_20_ values estimated using the most recent Italian data (i.e., in 2018) were lower than those calculated in 2010, approximately −4% above age 40 years in men. In women, the gap gradually increased with age: −2% at 40 years, −3% at 50 years, and −6% at 75 years. *R*_20_ values based on SEER data (i.e., those provided by ComPrev) were consistently lower than those calculated from Italian data for women but higher in men above age 30 years (**Table 6**).

### 3.6 Cure fraction and long-term NS

In **Table 7**, CF estimated by mixture cure models until the asymptotical time after diagnosis (thus age) was compared with the estimated 50-year NS and with NS until the attained age of 100 or 90 years, by cancer type, sex, and age at diagnosis.

**Table 7:** Model-based estimates of cure fraction (CF, %) (centered at 2010 as the year of diagnosis), net survival (NS, %) 50 years after diagnosis, until 100 years of age, and until 90 years of age, for selected cancer types by sex and age at diagnosis.

Cancer type (sex)	CF	NS until		
Age at diagnosis (years)		50 years after the diagnosis	Age 100 years	Age 90 years
All cancers (men)				
0–14	76%	79%	79%	79%
15–44	72%	77%	77%	77%
45–54	51%	56%	56%	57%
55–64	46%	53%	53%	53%
65–74	41%	48%	49%	50%
75+	35%	37%	38%	41%
All cancers (women)				
0–14	79%	81%	81%	81%
15–44	75%	79%	79%	79%
45–54	68%	72%	72%	72%
55–64	55%	60%	61%	62%
65–74	44%	48%	50%	52%
75+	34%	36%	36%	39%
Breast (women)				
0–44	72%	77%	77%	77%
45–54	77%	82%	82%	82%
55–64	71%	77%	77%	78%
65–74	61%	69%	72%	76%
75+	47%	51%	60%	72%
Prostate (men)				
0–44	68%	78%	78%	78%
45–54	86%	93%	93%	93%
55–64	89%	95%	95%	95%
65–74	81%	91%	93%	94%
75+	59%	68%	73%	80%

For pediatric cancer patients overall (age 0–14, **Table 7**), the difference between CF and 50-year NS is approximately 3%, suggesting a persistent excess risk of death throughout life, though limited. For the other patients, the difference was higher when diagnosed at ages 15–44 and 45–54 years (4%–5%). For older ages, both CF and 50-year NS go far beyond the maximum patient’s life span, and their interpretation is fuzzy. For men diagnosed with cancer (all types) at age 65–74 years, CF (asymptotical) was 41%, while the estimated NS after 50 years (attained age over 115 years) was 48%, 49% at the reached age of 100 years, and 50% at the reached age of 90 years. Differences were similar in women aged 65–74 years after any cancer type and after breast cancer (i.e., CF was 61%, 50-year NS was 69%, NS until 100 years was 72%, and NS until 90 years was 76%) (**Table 7**). Notably, patients diagnosed with prostate cancer at age ≥75 years had a CF = 59%, but the 50-year NS = 68%. The NS until 100 years was even higher (73%) and was 80% until 90 years.

### 3.7 Cure prevalence (CurePrev): examples and interpretation

**Figure 6:**
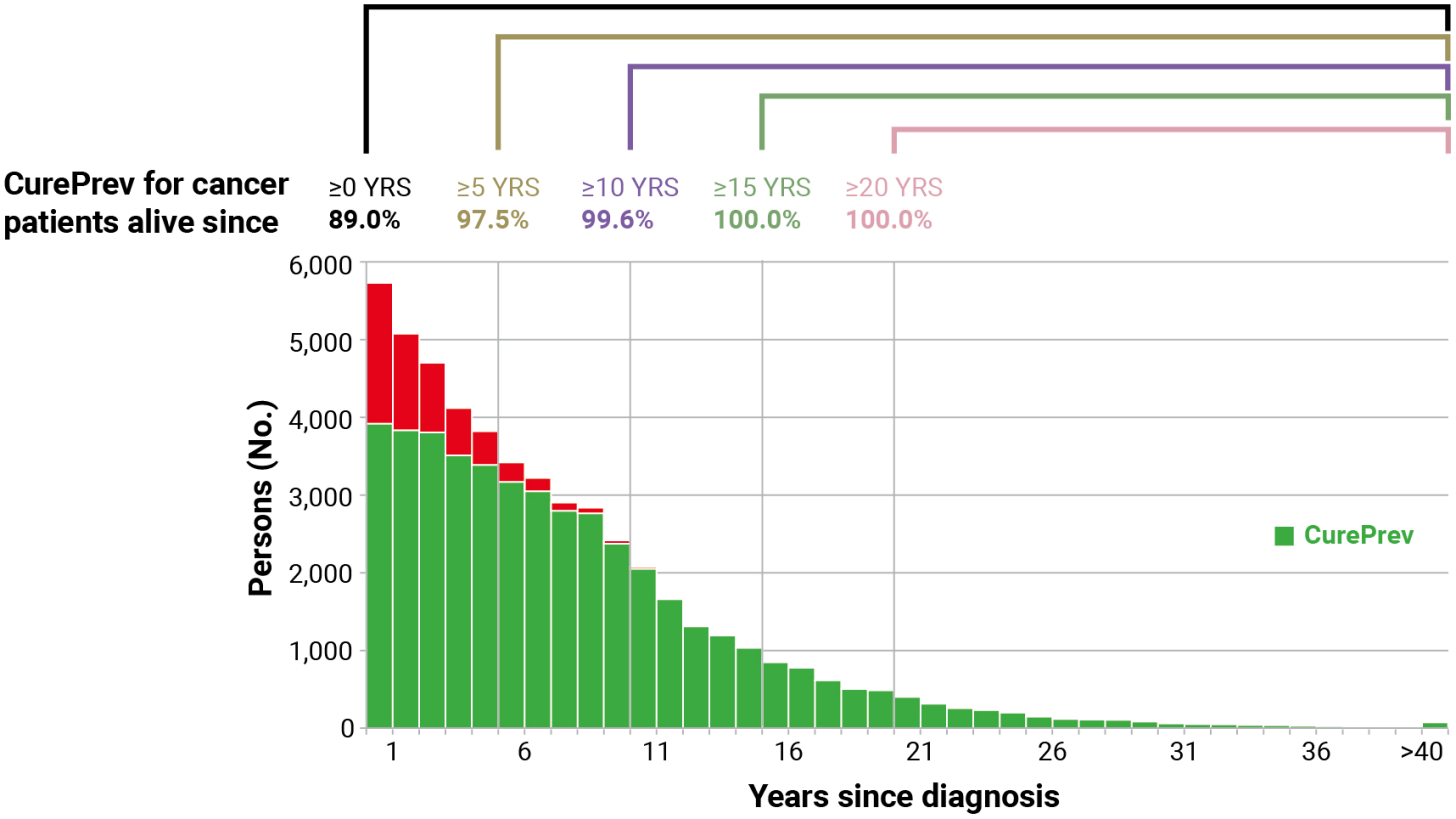
Cure prevalence (CurePrev) for Italian colorectal cancer patients (men and women), aged 55–64 years who were alive in 2018 (January 1st), overall and conditioned to be alive after more than 5, 10, 15, and 20 years. The red part of each bar includes cases who are expected to die because of their cancer.

The number of patients with colorectal cancer alive in 2018 (January 1st) at age 55–64 years has been presented in **Figure 6** (51,855 in the study area, sum of all bars). The green part of the bars included those expected to be cured, with the same mortality as the general population. CurePrev was 68.5% in those with diagnoses after ≤1 year (i.e., *CurePrev*(1) or the green area in the first vertical bar). CurePrev became 75.6% when diagnoses were >1 year and ≤2 years (i.e., the green area in the second vertical bar), and so on. The sum of CurePrev in all the annual intervals (vertical bars, overall CurePrev) was 89.0% and represented the proportion of colorectal cancer prevalent cases at age 55–64 years that will be cured (i.e., they will not die because of the neoplasm). Notably, the sum of *CurePrev*(*x*) for a duration longer than *t* years after diagnosis can be calculated as the sum of cases in green areas divided by all prevalent cases after a certain number of years (**Figure 6**). These *CurePrev* are the probabilities of being cured, conditioned to be already survive *t* years, and the complement of these quantities (i.e., 1 – *CurePrev*) can be read as the residual risk of death for cancer patients.

*CurePrev* for patients alive >5 years after diagnosis was 97.5% (i.e., 2.5% will die because of the neoplasms), 99.6% for patients alive after >10 years, and became 100.0% for those alive >15 years after diagnosis.

### 3.8 Already cured prevalence: examples

The same distribution of prevalent patients presented in **Figure 6** allowed also the estimation of patients who were already cured, that is the sum of patients alive more than 6 years after diagnosis or 48% of all colorectal cancer patients alive in 2018 at age 55–64 years (**Figure 4**). Notably, using the TTC (i.e., 7 years) calculated in the same age group of prevalent cases (attained age) (4), the proportion of *Prev*(>*TTC*) would be slightly underestimated, reaching only 42%.

## 4 Discussion

This study provides further insight into the models and procedures useful for estimating the number of people alive after a cancer diagnosis and several indicators of cancer cure. The validations presented describe reliable methods that can also be reproduced in different settings (i.e., countries).

According to our validations, some main observations deserve to be emphasized. The first one is on survival models, the basis for both the calculation of completeness indexes and cure indicators. Although the criteria for selecting the best model are still debated (25, 32), differences among the proposed parametric distributions to estimate long-term survival (e.g., non-mixture models, lognormal, flexible models with splines) (6, 14, 33) are limited (32) when sufficient population size and long follow-up are available. In addition, model-based age-stratified estimates based on Weibull distribution of fatal cases showed a very good fit with “observed” net survival for common cancer types (i.e., breast or colorectal at any age and stage and prostate) (**Supplementary Figures 2–4**) and support their use to estimate completeness index and complete prevalence, as well as cure fraction and time to cure.

A second observation concerns our validation of the impact on the complete prevalence of using different completeness indexes. In principle, models should be built from complete and homogeneous registration periods (i.e., generally short) and, at the same time, should capture long-term survival and incidence trends (i.e., preferably long). Our validations show that the more accurate behavior of completeness indices was obtained using long-term incidence and survival data, although not all CRs provide data for all the years in the study period (**Table 4**). These results are explained by the assumptions of the completeness index method, calculated by including a back-estimation of incidence before the observed period through age-cohort models, assuming there is no period effect, although often very pronounced (e.g., for prostate after PSA diffusion, after breast cancer screening, for thyroid cancer). This observation may support similar choices in other countries (34) and suggests that more accurate complete prevalence estimations may be obtained using completeness indexes calculated from countries or regions with patterns (e.g., absolute values of incidence and survival and trends of incidence) similar to those of the registry or area to which they will be applied.

A third point worthy of discussion concerns the assumptions and interpretations of the cure fraction, the estimation of which is also sensitive to the statistical model used. The population-level cure can be estimated by cure models assuming that there are two groups of patients: a group of individuals who experience no excess mortality, whose proportion is estimated by the cure fraction parameter, and a second group (i.e., uncured cases) who experience excess mortality that follows a survival function (35). Cure at the population level is a reasonable and widely accepted hypothesis when the net survival curves plateau and the excess mortality rate was negligible at some point within the follow-up interval (25). When excess mortality estimates (i.e., net survival) show a non-negligible decrease until the maximum follow-up time, the cure fraction should be read only as the proportion of diagnosed cancer patients that will die for causes other than their specific cancer (5), even if we know nothing about the time when those people will die. In the present study, we compared for the first time the estimates of the widely used “asymptotical” cure fraction (which are based on extrapolating very distant observations for periods beyond the end of available follow-up) and estimates of net survival until a reasonable maximum age that a patient may reach (i.e., until age 90 or 100 years, the long tail of the modeled NS curve). The difference between CF and 50-year NS in childhood cancer patients (3% in men and 2% in women), as well as in young adults (15–44 years, 5% and 4%, respectively), should be highlighted, in agreement with studies showing an excess risk of childhood cancer patients for many years after diagnosis (i.e., throughout life) due to treatment effects, second malignancies, or host features (36, 37). The same difference is still more marked for older patients. However, from the patient’s point of view and to apply this information to clinical surveillance, it does not seem useful to consider a pediatric patient as uncured when they are alive several decades after diagnosis (38), or if she/he is still alive at age 100 years with a small excess risk of death.

In general, it should be noted that the assumption of only two groups of patients (i.e., cured and uncured), aside from being an extreme simplification, is very conservative. Some patients may have a risk of death higher than the general population associated with the same genetic background, lifestyle, and environmental factors associated with cancer diagnosis (39). The mixture cure models used in this paper did not include the patients’ increased deaths from other causes that can be directly related (e.g., adverse effects of treatments) or not (e.g., independent second cancer) with the studied cancer, compared to the general population. Disregarding the presence of this factor leads to estimating a lower proportion of cures, given the definition of cures as those patients who will not die from relapse or disease progression (40). Younger patients, in particular, may be exposed to the detrimental effects of cancer treatments. To overcome these limitations, a more complex mixture model was proposed to capture not only cured and not cured but also the long-term risk of death in children diagnosed with cancer, due to the side effects of cancer treatments, second cancers, and risk factors associated with first cancer carrying an extra risk of death for patients (41). These models should be extended and validated also in adults.

A final point to be highlighted is the calculation and interpretation of cure prevalence, an indicator of the proportion of patients that have the same life expectancy as individuals in the general population of the same sex and age (4, 15). As the number of years since diagnosis increases (conditional on survival). This indicator can be read as the complement of the residual probability of dying from cancer (conditioned to be already survived) and can be helpful to overcome the difficulties of cancer survivors in accessing insurance for a home loan or a mortgage (42, 43).

### 4.1 Strengths and weaknesses

The major strengths of the presented study are the comprehensive description of the following issues: how the different completeness indices may impact the calculation of complete prevalence, the calculation of indicators of cure with the improvement of algorithms used, and the formal exposition of the links among the different indicators. In the estimation of already cured prevalence, we applied to prevalent cases at attained age the TTC calculated at the age of diagnosis, overcoming the simplified assumption used in the past, when TTC was applied to the complete prevalence of more advanced (reference) ages (4), an assumption that could lead to a slight underestimation of indicator since the TTC increased with age for most of the cancer types. The completeness and accuracy of the Italian CR incidence and survival data were deemed satisfactory (1, 44) and represent a major strength of the study, in particular for the estimation of long-term survival, cure, and prevalence. In addition, the size of the study population and the follow-up length (≥15 years for all CR used in the modelization) contributed also to maximize the reliability of the estimates of incidence and survival parameters, and indicators of cure. It should be noted that few CRs have the last available incidence year and LDP before 2017. For them, LDP and CP (not incidence or survival) were projected in 2018 and thereafter. In our medium-term projections, the hypothesis that CP can be predicted by a linear function of the calendar year as a regressor variable is supported by empirical evidence, at least for all cancer types combined and for most frequent cancer types, consistently showing an approximately linear trend in recent years (17, 22, 45).

Our study has some limitations. First, the probabilities of death for a cause (cancer *vs*. other causes) are estimated at the population level. Therefore, they reflect the overall behavior of a population, which may differ among individuals with cancer (i.e., an individual with comorbidities whose other cause of mortality might be greater or an individual who is compliant with cancer screening programs and whose high health awareness may result in lower other-cause mortality than the general population) (46). Second, in our study, we used an *a priori* threshold of 5% (of 5-year CNS) as a threshold of a low risk of death from cancer, which may be relatively unrestrictive for some groups and inevitably arbitrary. Sensitivity analyses were performed varying this threshold as well as different definitions were used (3, 6, 7, 10). A lower cutoff may be useful among younger individuals who are at low risk of death from other causes (10), and when years to reach 5- or 10-year CNS >90% or 95% were explored (4). It should be noted that the estimation of TTC is sensitive to the choice of the CNS threshold (i.e., 90% or 95% to fix a low risk of recurrence/death or the margin of clinical relevance) and the methodological approach used (3, 4, 7, 8, 10, 32), in particular for cancer types with a non-negligible long-term excess mortality rate (e.g., prostate or breast cancer). Nevertheless, the 5-year CNS >95% is not only clinically relevant and widely reproducible, but it also allows comparability between countries (5, 32, 47, 48).

In addition to the fact that estimates of cure indicators are sensitive to the different models used (whose choice has less impact on the calculation of the completeness index), a specific limit of the present study is that only mixture cure models parametrized according to Weibull or exponential distributions are allowed by the ComPrev software (30). Our mixture model was designed to capture only the long-term excess risk of death due to cancer. The advantages of alternative models include greater modeling flexibility as regards the shapes of the survival distributions and greater sensitivity to small excess risk (14, 33).

Another limitation of studies performing epidemiological indicator projections (17, 49) is the evolution of demographic trends (fertility, migration, and life expectancy) which have a strong impact on predictions of the future population at risk of cancer and profoundly affect the future burden of the cancer prevalence. For instance, the Italian population in 2020 observed in 2022 was 59.6 million, while the same population forecasted in 2015 (17) was 62.5 million (+5%), leading to an overestimation of the absolute number of prevalent cases.

Finally, it should be emphasized that net survival estimates, as cure models, are less reliable for older age groups (e.g., 75 years or more). It is, however, very useful to calculate prevalence (and related indicators) at all ages even if certain cure indicators (i.e., CF and TTC) are considerably less reliable (as well as possibly less useful) for older patients.

## 5 Conclusions

In the context of a population of cancer survivors expected to increase significantly in Europe and other high-income countries (45, 49, 50), this paper represents an important addition to the current knowledge on the topic providing a comprehensive picture of several available indicators of prevalence and cancer cure. They are unambiguously defined, measurable, and reproducible, e.g., the estimation of the same indicators can be performed in different countries and periods in areas with coverage by population-based cancer registries. Although cure fractions and time to cure are appealing in a clinical context and have widespread applicability, estimation relies on several choices, each associated with pitfalls, that the practitioner should be aware of (30, 43). Nevertheless, these indicators may help to better categorize cancer patients according to the risk of relapse or death many years after diagnosis (12, 51).

## Data availability statement

Research data (aggregate) are available from the corresponding authors upon reasonable request.

## Ethics statement

Ethical review and approval was not required for the study on human participants in accordance with the local legislation and institutional requirements. Written informed consent from the participants’ legal guardian/next of kin was not required to participate in this study in accordance with the national legislation and the institutional requirements.

## Author contributions

LDM and SG drafted the study protocol and the other authors revised the study protocol, collected the data, and prepared the cleaned data for the study database (SFr, RDA, DS, MZ, GM, EB, AR, FC, EM, AP, MFe, CG, MG, GCar, FS, MaM, RC, WM, MFu, PB, GS, SFe, LM, RR, MiM, GCas, LoB, RG, DP, MP, FB, PS, AF, and PP). FT, SG, ADP, and LDM designed the study and did the statistical analyses. SF, RDA, EC, LaB, SR, and SM contributed to the validation of statistical models and revised the statistical analyses. EC and DS specifically discussed the assumptions and clinical implications of the indicators of cancer cure. All authors contributed to the interpretation of the study results. All authors contributed to the article and approved the submitted version.

## Members of AIRTUM Working Group

Fabiola Giudici, Ellina Evdokimova (CRO Aviano), Elena Demuru (ISS Roma), Gemma Gatta, Paolo Contiero, Giovanna Tagliabue (Fondazione IRCCS Istituto Nazionale Tumori Milano), Riccardo Capocaccia (E&P), Massimo Rugge (Veneto Cancer Registry–CR), Teresa Intrieri (Tuscany CR), Martina Taborelli (Friuli Venezia Giulia CR), Lucia Bisceglia (AReSS Puglia CR), Stefano Rosso (Piedmont Cancer Registry), Claudia Casella (Liguria CR), Antonietta Torrisi (Catania-Messina-Enna CR), Giovanni Maifredi (Brescia CR), Monica Lanzoni (ATS Insubria CR), Alessio Gili (Umbria CR), Sergio Mazzola (Palermo CR), Maria Francesca Vitale (Napoli 3 Sud CR), Erica Giacomazzi (Val Padana CR), Silvia Ghisleni (Bergamo CR), Maria Adalgisa Gentilini (Trento CR), Fabio Vitadello (SABES-ASDAA Cancer Registry; IRTS), Concetta Patrizia Rollo (Ragusa-Caltanissetta CR), Stefano Marguati (Pavia Cancer Registry), Luciana Del Riccio (Basilicata CR), Maria Rotella (Nord Sardegna CR), Alessandra Sessa (Caserta CR), Antonino Colanino Ziino (Siracusa CR), Ivan Cometti (Sondrio CR), Roberta Bosu (Nuoro CR).
