# Supplementary material for: Complete prevalence and indicators of cancer cure: enhanced methods and validation in Italian population-based cancer registries
Source: Front Oncol. 2023 Jun 6;13:1168325. doi: 10.3389/fonc.2023.1168325 (PMC10280813; doi:10.3389/fonc.2023.1168325)
Supplement: Supplementary file 1 [file DataSheet_1.docx]

**Supplementary Table 1. Best fitting survival models used by cancer type**

| **Site or type** | **Model used (age stratification)^1^** |
| --- | --- |
| All cancers but skin | Weibull, age stratified |
| Head and neck | Weibull, age stratified**^2^** |
| Oral cavity | Weibull, age stratified**^2^** |
| Mouth (except base of Tongue) | Weibull |
| Salivary glands | Exponential, age stratified^4^ |
| Oropharynx | Weibull |
| Nasopharynx | Weibull |
| Esophagus | Weibull, age stratified |
| Stomach | Weibull, age stratified |
| Small intestine | Exponential, age stratified |
| Colorectal | Weibull, age stratified |
| Colorectal, stage I | Exponential, age stratified^*^ |
| Colorectal, stage II | Exponential, age stratified |
| Colorectal, stage III-IV | Weibull, age stratified |
| Colon | Weibull, age stratified |
| Rectal | Weibull, age stratified |
| Anus | Weibull, age stratified |
| Liver | Weibull, age stratified |
| Hepatocellular carcinoma | Weibull |
| Intrahepatic cholangiocarcinoma | Weibull, age stratified |
| Other hepatic cancer | Weibull, age stratified |
| Gallbladder | Weibull, age stratified |
| Pancreas | Weibull, age stratified |
| Larynx | Exponential, age stratified |
| Lung bronchus trachea | Weibull, age stratified |
| Bone | Weibull |
| Skin melanoma | Weibull, age stratified |
| Mesothelioma | Weibull, age stratified |
| Kaposi sarcoma | Exponential, age stratified^3^ |
| Connective tissue | Weibull |
| Soft-tissue sarcoma | Weibull |
| Bone sarcoma | Weibull |
| GIST | Weibull^4^ |
| Breast (Women only) | Weibull, age stratified |
| Breast, stage I | Weibull |
| Breast, stage II | Weibull, age stratified |
| Breast, stage III-IV | Weibull, age stratified |
| Vagina and vulva | Weibull, age stratified |
| Vulvar SCC | Weibull |
| Cervix uteri | Weibull, age stratified |
| Corpus uteri | Weibull, age stratified |
| Ovary | Weibull, age stratified |
| Penis | Exponential, age stratified |
| Prostate | Weibull, age stratified |
| Testis | Weibull |
| Kidney | Weibull, age stratified |
| Bladder | Weibull |
| Brain and CNS | Weibull, age stratified |
| Thyroid | Exponential, age stratified |
| Thyroid, papillary | Exponential, age stratified^4^ |
| Thyroid, follicular | Exponential, age stratified^4^ |
| Thyroid , anaplastic | Weibull |
| Thyroid, medullary | Exponential, age stratified^4^ |
| Hodgkin lymphoma | Exponential, age stratified^1^ |
| Non-Hodgkin lymphoma | Exponential, age stratified^1^ |
| CLL/SLL | Weibull, age stratified**^4^** |
| NHL, DLBC | Weibull, age stratified**^4^** |
| NHL, follicular | Exponential, age stratified**^4^** |
| Myeloma (Plasma Cell) | Exponential, age stratified |
| Leukemia | Exponential, age stratified |
| precursor cell acute lymphoblastic leukemia | Weibull, age stratified |
| acute myeloid leukemia | Weibull, age stratified |
| chronic myeloid leukemia | Exponential, age stratified |

**^1^** Age groups were 0-44,45-54-55-64,65-74,75+, except for All cancers but skin, Brain and CNS, Hodgkin lymphoma, Non-Hodgkin lymphoma, Leukaemia, precursor cell acute lymphoblastic leukemia, acute myeloid leukemia (0-14, 15-44, 45-54, 55-64, 65-74, 75+), and SLL/CLL (0-54,55+). **^2^** Parameters in women from men+women estimates.

^3^ Parameters in women, from men estimates. **^4^** Parameters in women and in men, from men+women estimates.

**Supplementary Figure 1. Flowchart of the calculation of complete prevalence and indicators of cure.**

**
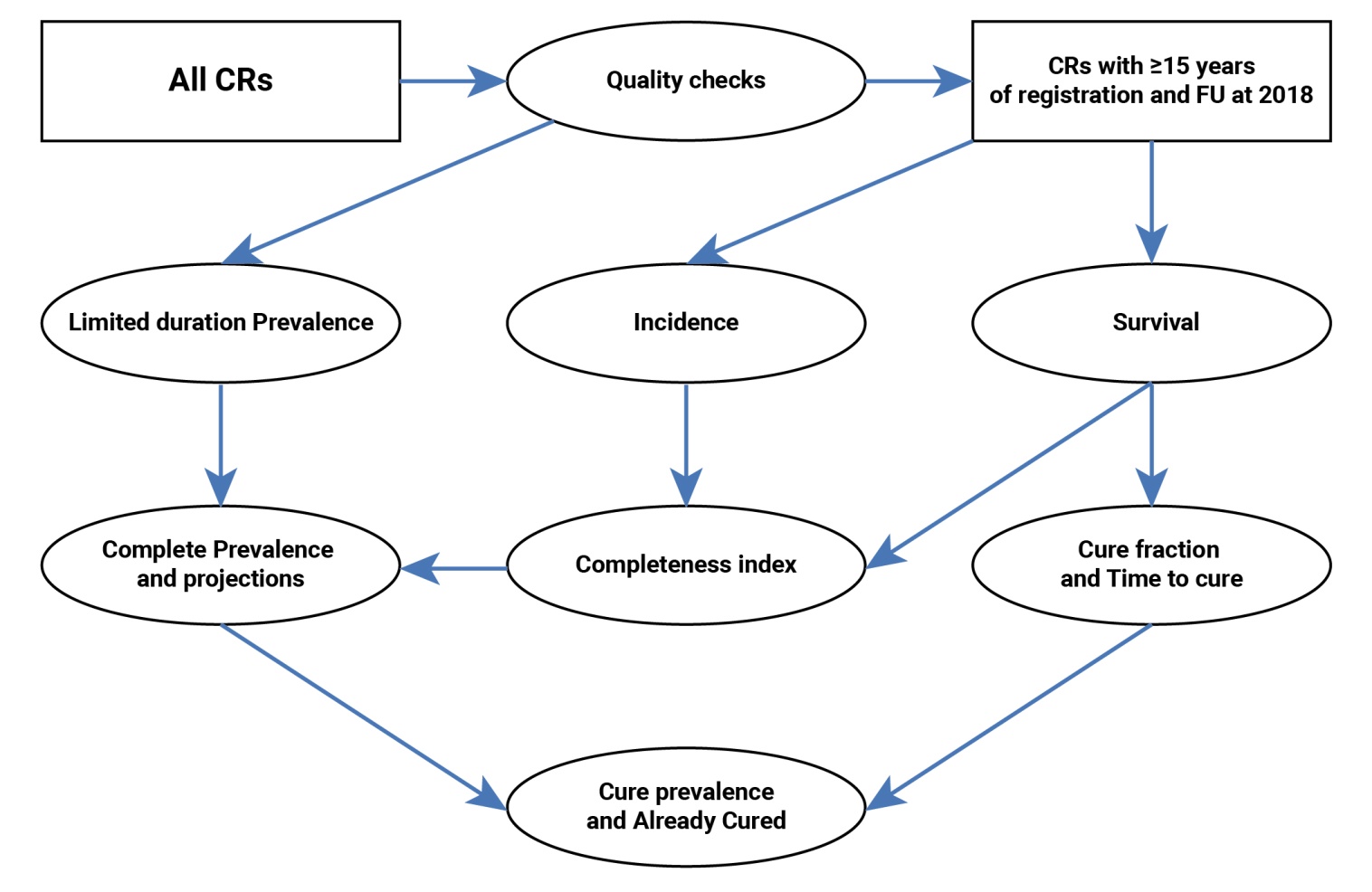
**

CRs: Cancer Registry, FU: follow-upupp1Flow20221115.pptx

**Supplementary Figure 2. Net Survival (NS), 5-year conditional NS (5-yr CNS), and corresponding model-based estimates until 20 years of follow-up for breast cancer patients aged 55-74 years, diagnosed in 1997-1999 and followed-up until 2018, by stage**


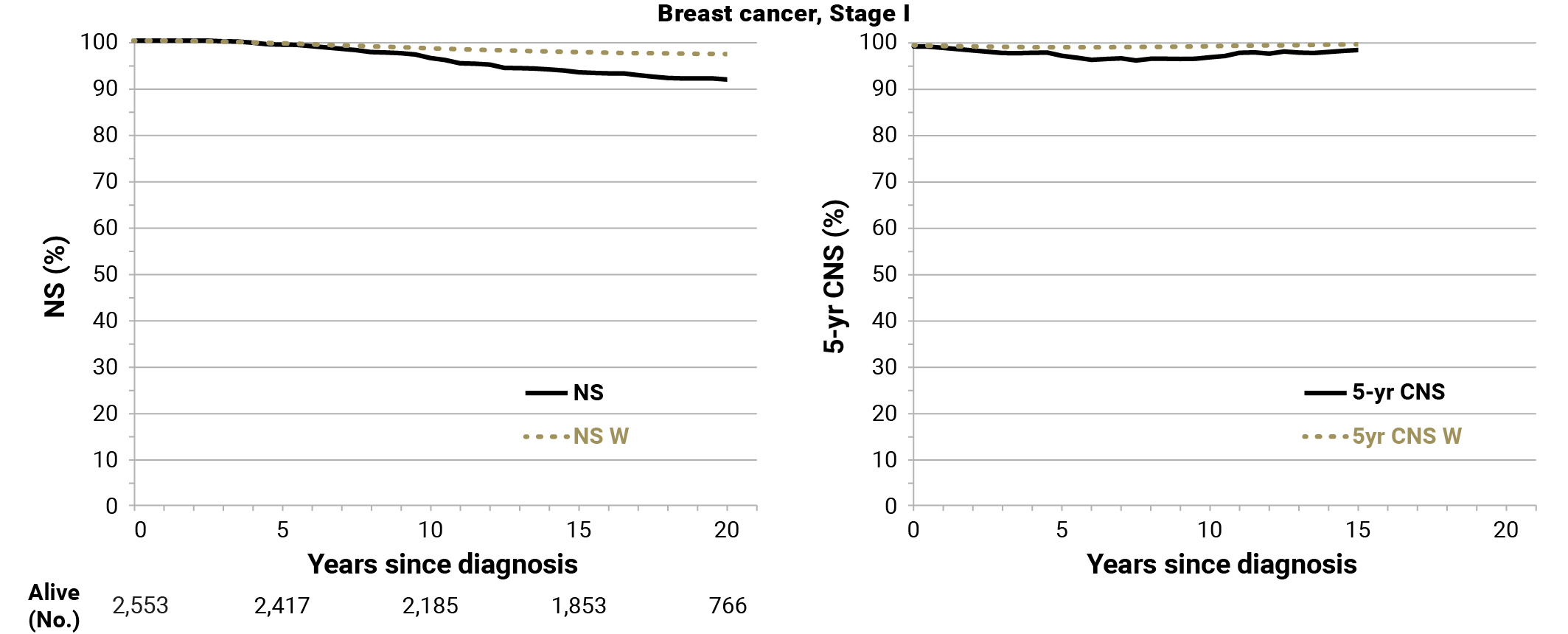


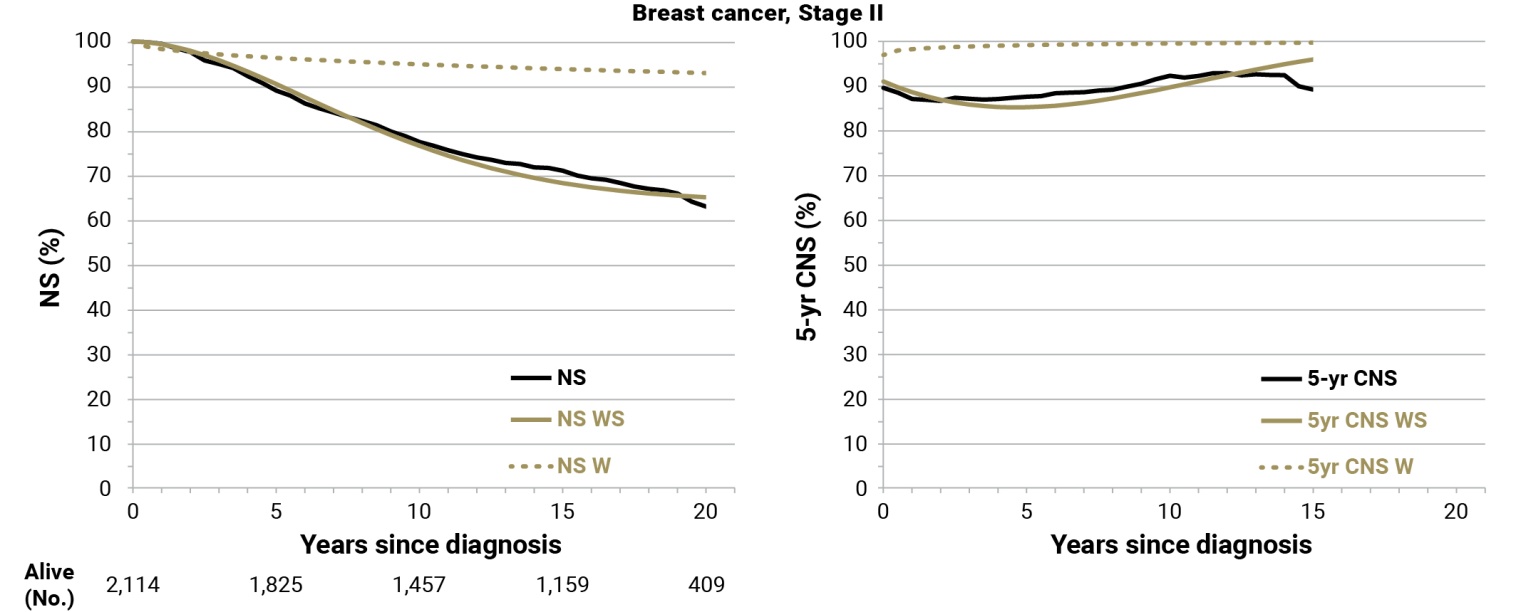


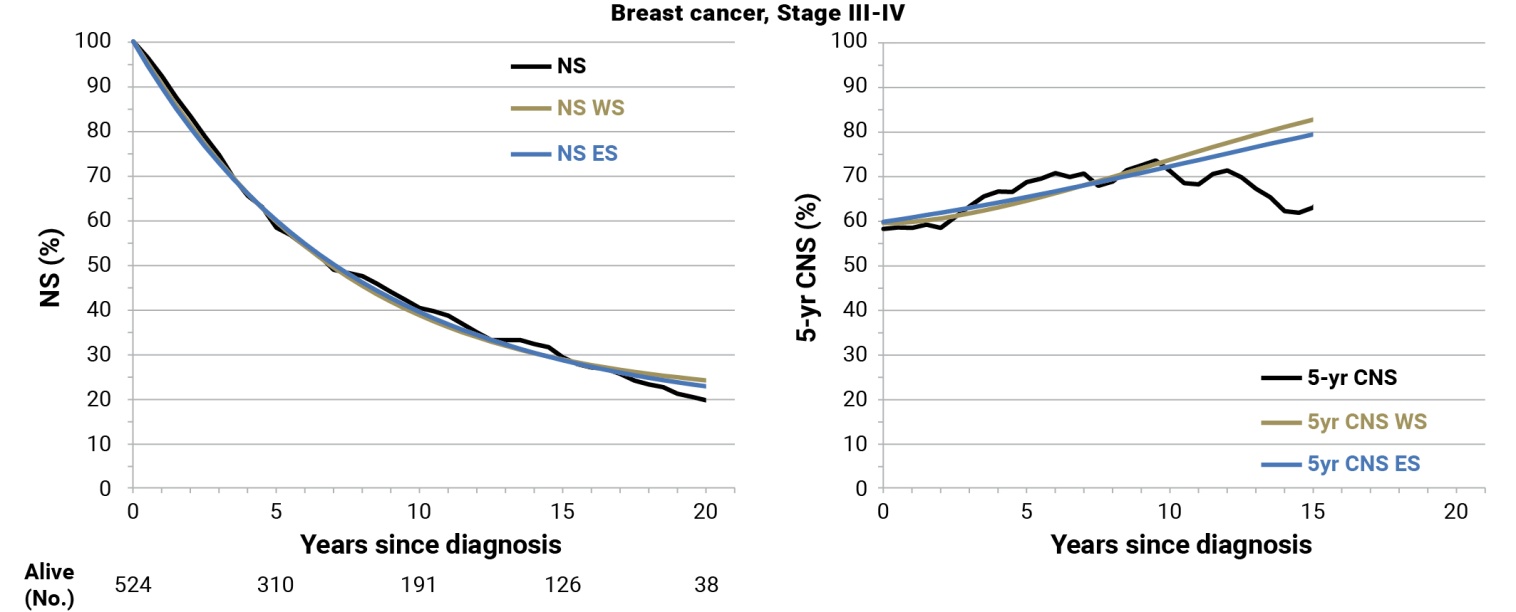


W = Weibull; WS = Weibull, Age-Stratified; E = Exponential; ES = Exponential, Age-Stratified

**Supplementary Figure 3. Net Survival (NS), 5-year conditional NS (5-yr CNS), and corresponding model-based estimates until 20 years of follow-up for colorectal cancer patients (men and women) aged 65-74 years, diagnosed in 1997-1999 and followed-up until 2018, by stage**


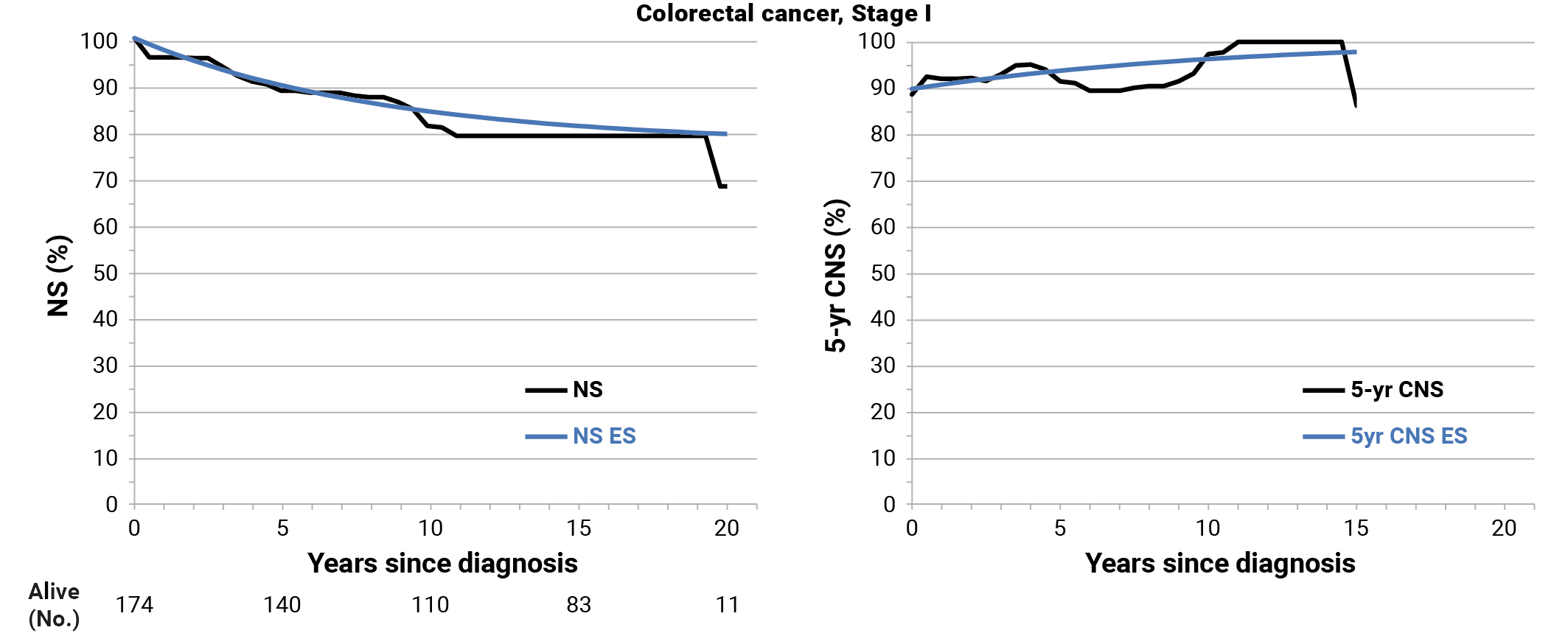

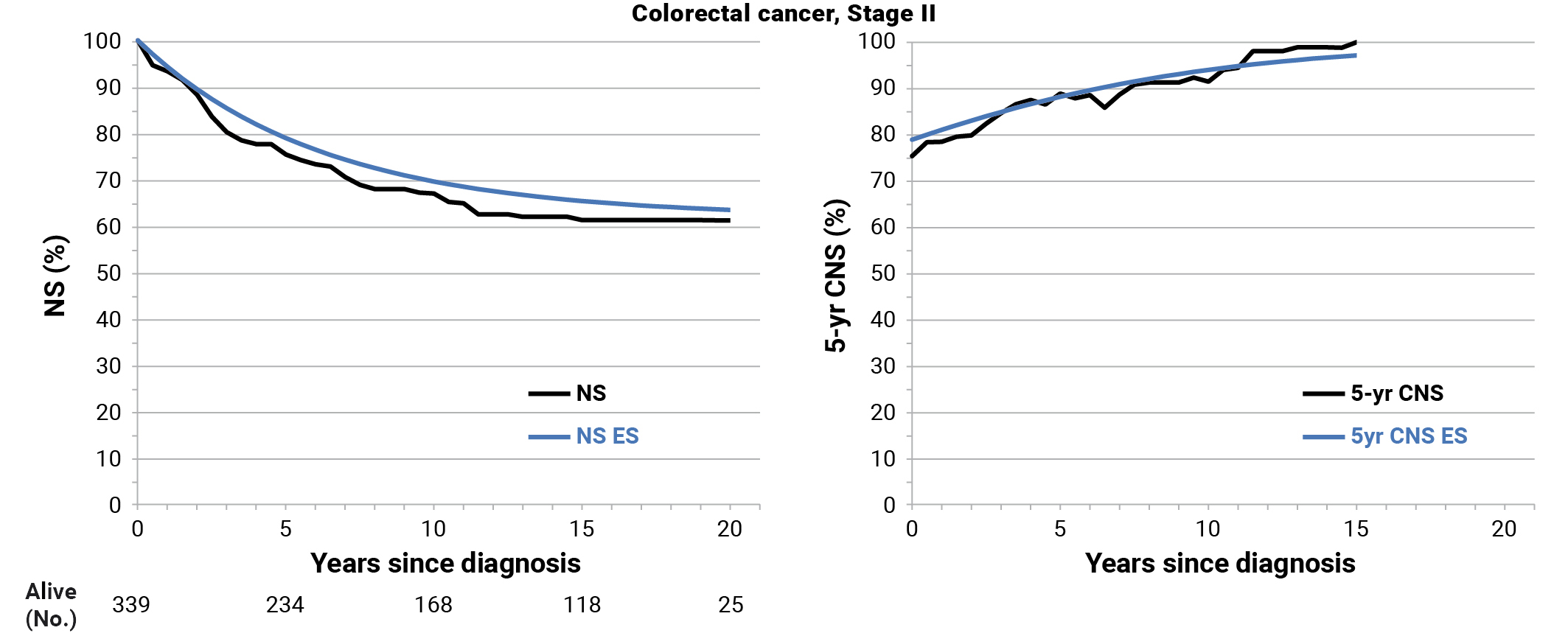

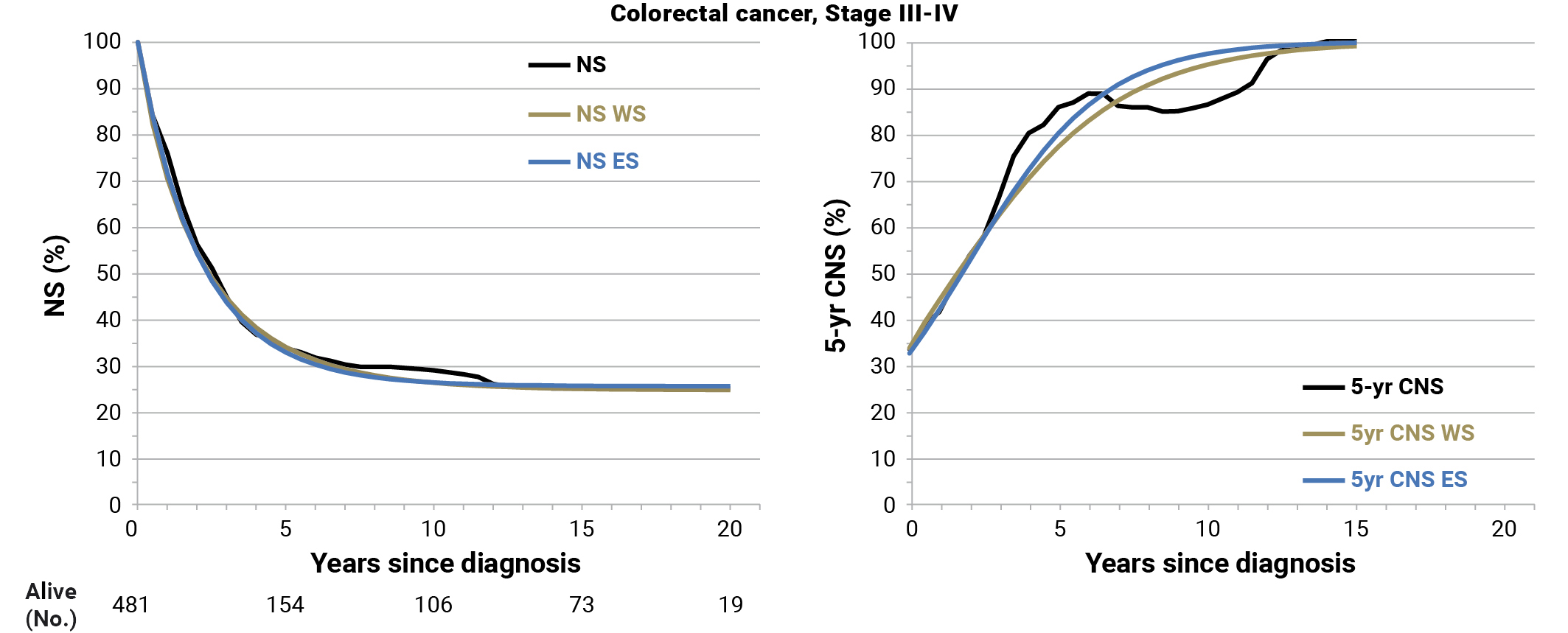


W = Weibull; WS = Weibull, Age-Stratified; E = Exponential; ES = Exponential, Age-Stratified

**Supplementary Figure 4. Net Survival (NS), 5-year conditional NS (5-yr CNS), and corresponding model-based estimates until 14 years of follow-up for patients with prostate cancer and soft tissue sarcoma diagnosed in 2003-2005 and followed-up until 2018**


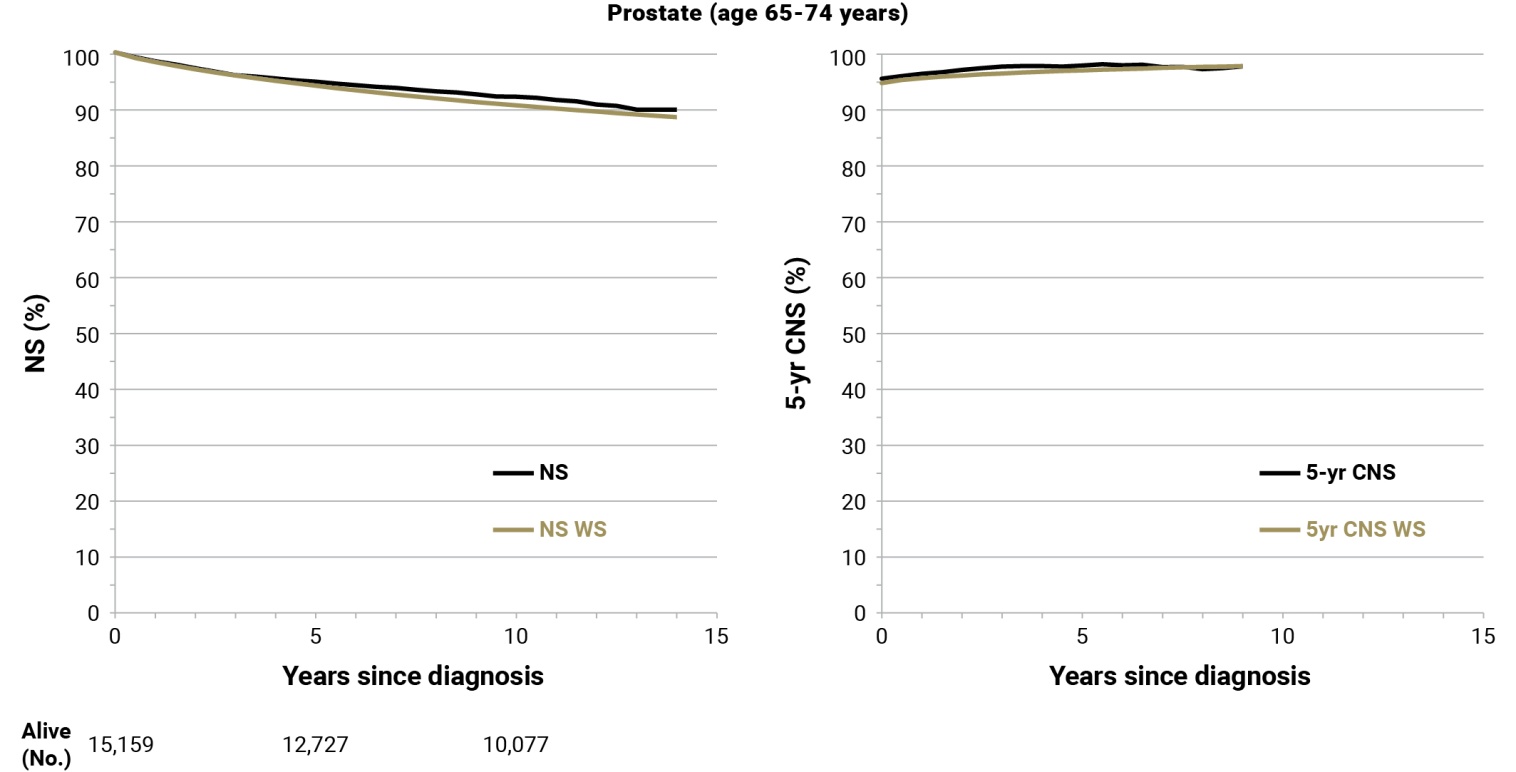


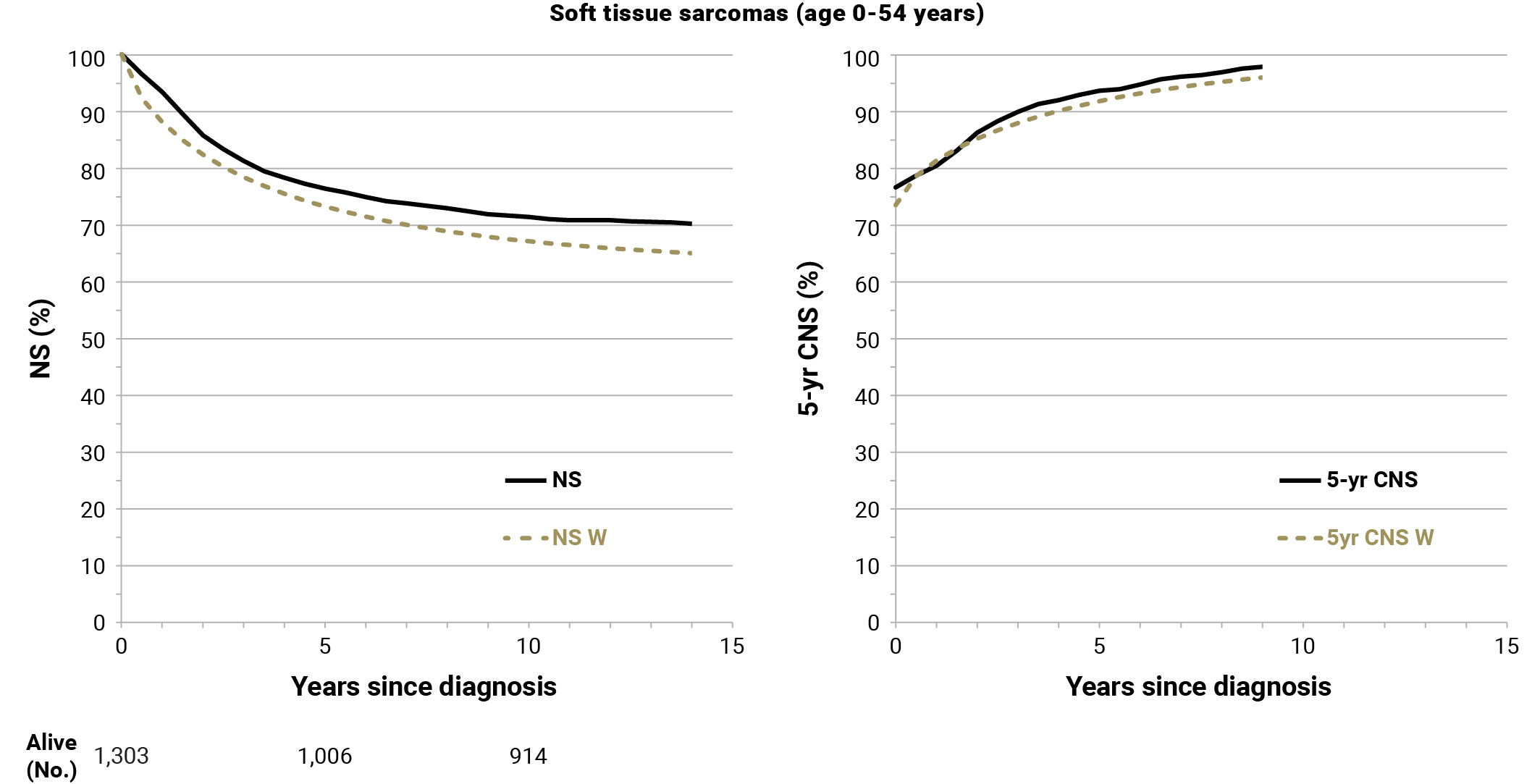


W = Weibull; WS = Weibull, Age-Stratified; E = Exponential; ES = Exponential, Age-Stratified

**Supplementary Figure 5. Observed and model-based^1^ age-specific incidence rates for all cancers combined, prostate and breast cancer. Italy 1990-2014**

^
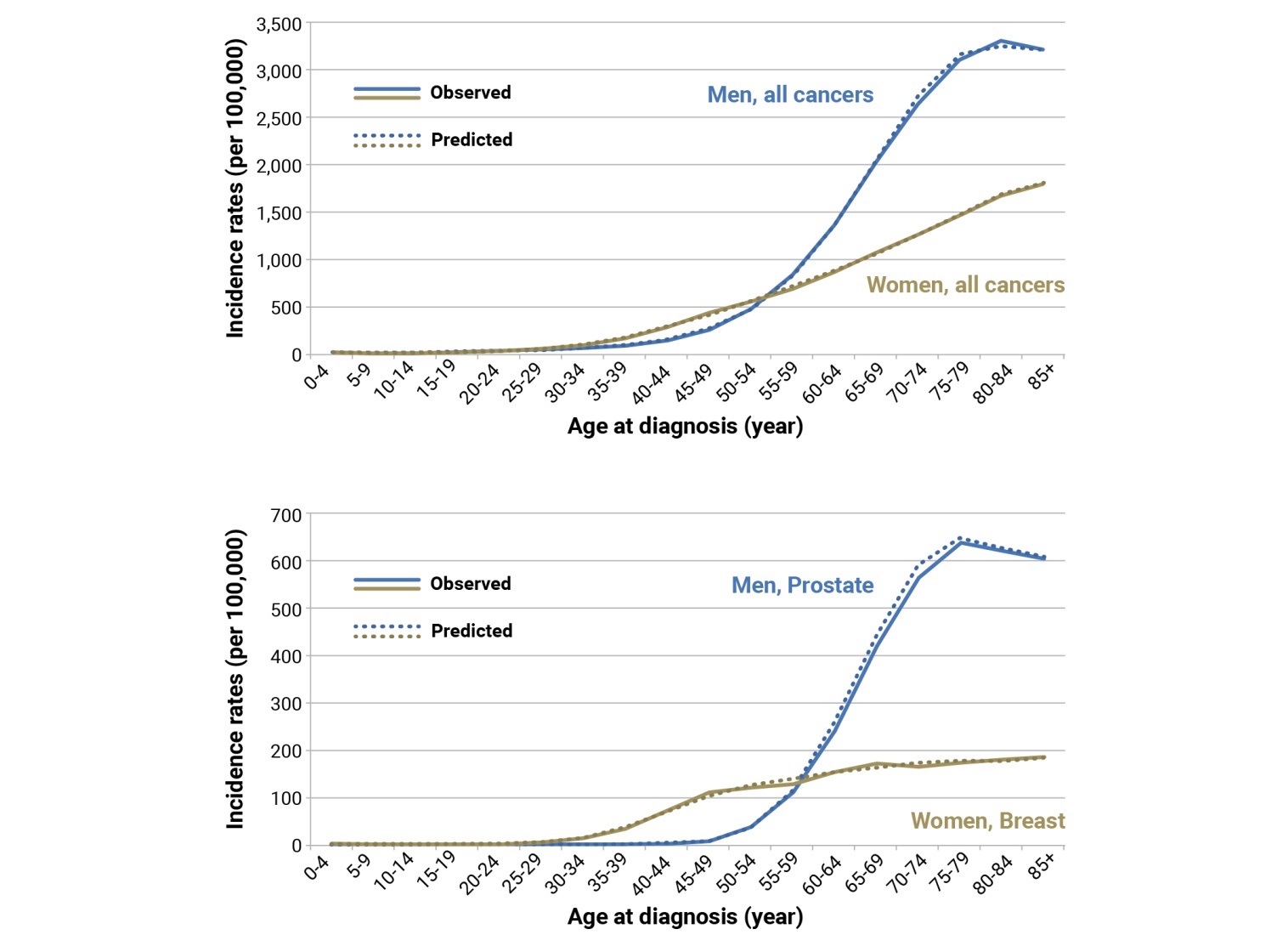
1^ According to a sixth degree polynomial on age.
